# Molecular endotyping in people with bronchiectasis based on response to antibiotic treatment: iBEST study

**DOI:** 10.1183/23120541.00389-2025

**Published:** 2025-12-29

**Authors:** Gisli G. Einarsson, Laura J. Sherrard, Andrew J. Lee, Jack Carson, Andrew Marshall, Aya Alkhatib, Vanessa Brown, Deirdre F. Gilpin, Gerhild Angyalosi, Michael R. Loebinger, James D. Chalmers, Francesco Blasi, Charles S. Haworth, Eva Polverino, Harm A.W.M. Tiddens, Herman Goossens, Felix C. Ringshausen, Adam T. Hill, J. Stuart Elborn, Michael M. Tunney

**Affiliations:** 1 Queen's University Belfast, Belfast, UK; 2 Halo Research Group, School of Pharmacy, Queen's University Belfast, Belfast, UK; 3 Centre for Experimental Medicine, Queen's University Belfast, Belfast, UK; 4 Novartis Pharma AG, Basel, Switzerland; 5 Host Defence Unit, Royal Brompton Hospital and Harefield NHS Foundation Trust, London, UK; 6 Imperial College London, London, UK; 7 Scottish Centre for Respiratory Research, University of Dundee, Ninewells Hospital and Medical School, Dundee, UK; 8 Internal Medicine Department, Respiratory Unit and Adult Cystic Fibrosis Center, Fondazione IRCCS Cà Granda Ospedale Maggiore Policlinico, Milan, Italy; 9 Department of Pathophysiology and Transplantation, Università degli Studi di Milano, Milan, Italy; 10 Cambridge Centre for Lung Infection, Royal Papworth Hospital NHS Foundation Trust, Cambridge, UK; 11 Department of Medicine, University of Cambridge, Cambridge, UK; 12 Respiratory Disease Department, Vall d’ Hebron University Hospital – VHIR, Barcelona, Spain; 13 Dept of Paediatric Pulmonology and Allergology, Erasmus Medical Centre Sophia Children's Hospital, Rotterdam, The Netherlands; 14 Dept of Radiology and Nuclear Medicine, Erasmus Medical Centre, Rotterdam, The Netherlands; 15 Dept of Clinical Microbiology, University Hospital Antwerp, Antwerp, Belgium; 16 Dept of Respiratory Medicine, Hannover Medical School, and Biomedical Research in End-stage and Obstructive Lung Disease Hannover (BREATH), German Center for Lung Research (DZL), Hannover, Germany; 17 Dept of Respiratory Medicine, Royal Infirmary of Edinburgh, and University of Edinburgh, Edinburgh, UK; 18 G.G. Einarsson and L.J. Sherrard contributed equally as first authors; 19 J.S. Elborn and M.M. Tunney contributed equally as senior authors

## Abstract

**Background:**

Culture-independent molecular techniques could potentially be used to measure microbiological efficacy in response to antibiotic treatment and improve understanding of the role of the airway microbiota in determining response in patients with chronic respiratory disease.

**Methods:**

Using molecular methods, we analysed changes in the sputum microbiota in samples from 107 participants with bronchiectasis recruited to the iBEST-1 study, and defined community endotypes based on response to tobramycin inhalation powder (TIP) treatment. The relationship between microbiota metrics in these endotypes and clinical and inflammatory biomarkers were also determined.

**Results:**

There was a significant reduction in *Pseudomonas aeruginosa* density, measured by quantitative polymerase chain reaction (qPCR), between Days 1 and 29 for participants in the TIP treatment (n=63; p<0.0001) but not placebo (n=20; p>0.05) group. Based on decrease in *P. aeruginosa* density (*oprL* copies·mL^−1^) over 28 days, two clusters of participants receiving TIP were observed and stratified as either responders (≥2Log_10_; n=26) or non-responders (<2Log_10_; n=37). In responders, a shift to a microbial community structure less dominated (p=0.018) by a pathogen was apparent and associated with a greater improvement in inflammatory and fewer participant exacerbations in the following 6 months (27% *versus* 49%; p=0.117) when compared to non-responders. Lung function was higher at Day 1 in responders (_median_=64.6% predicted) than non-responders (μ̃_median_=50.3% predicted) and independently predicted response to treatment with TIP (p=0.013).

**Conclusions:**

qPCR may be a useful, culture-independent microbiological efficacy end-point in clinical trials. Using qPCR, participants with bronchiectasis were stratified into endotpyes which predicted response to antimicrobial treatment, potentially allowing for a more personalised approach to therapy.

## Shareable abstract

**qPCR identifies bronchiectasis endotypes associated with antimicrobial response. TIP responders show reduced *P. aeruginosa*, improved inflammation and fewer exacerbations, highlighting the need for tailored therapy.** https://bit.ly/4lpAS3d

## Introduction

In patients with bronchiectasis (BE), chronic *Pseudomonas aeruginosa* infection is associated with increased exacerbation frequency, accelerated lung function decline, hospital admissions and increased mortality [1–3]. There is a direct relationship between bacterial load, airway inflammation and exacerbation risk supporting the use of long-term inhaled antibiotics [4, 5].

A microbiological secondary outcome is recommended for all trials of bronchopulmonary infection [6] which should include measures of bacterial density (*e.g.* colony-forming units per gram (CFU·g^−1^) of sputum) and resistance (*e.g.* minimum inhibitory concentration (MIC)). Traditional culture techniques are the gold standard for bacterial identification and enumeration, but they are time-consuming and labour intensive. Molecular diagnostic techniques, such as quantitative polymerase chain reaction (qPCR) and next-generation sequencing (NGS), are promising options to measure microbiological efficacy end-points including change in total bacterial density, density of specific pathogens and microbiota composition in response to treatment with anti-infective agents.

The iBEST-1 study assessed the safety and tolerability of tobramycin inhalation powder (TIP) in patients with BE; all three doses of TIP tested reduced *P. aeruginosa* density, measured by quantitative culture, from Day 1 to Day 29 *versus* placebo in a dose-dependent manner [7]. In the present study, we determined if molecular techniques could be used to measure the change in *P. aeruginosa* density following treatment with TIP *versus* placebo in iBEST-1 participants. Moreover, based on recent studies that have defined inflammatory and microbiota endotypes in BE [8–10], we hypothesised that participants could be stratified into endotypes based on response to antibiotic treatment.

## Methods

Further methodology details are provided in the online supplementary material.

### Study design and participants

The iBEST-1 study design and participant recruitment details are published [11]. Briefly, participants (n=107) were randomised in a 1:1:1:1 ratio to one of three TIP treatment cohorts (A, 84 mg tobramycin; B, 112 mg tobramycin; C, 224 mg tobramycin) or placebo for 16 weeks (Day 1 to Day 113) with a further 8-week follow-up period (Day 113 to Day 169). Participants received TIP, in either a continuous daily or cyclical regimen (alternating 28 days TIP/28 days placebo), or placebo.

The present study focused on participants who had paired sputum samples available at Day 1 and Day 29, as change in *P. aeruginosa* density (quantitative culture) between these time-points was the primary study efficacy end-point. All participants stratified to a treatment cohort, regardless of regimen, received TIP for the first 28 days (*i.e.*, Day 1 to Day 29). Participant characteristics recorded included sex, age, spirometry measurements and occurrence of pulmonary exacerbations. *P. aeruginosa* density by culture (CFU·g^−1^) and tobramycin MIC [7] determined previously were included.

### Sputa preparation

Extraction and purification of genomic bacterial DNA from sputa were performed on the MagNA Pure 96 automated DNA extraction platform.

### Determination of bacterial density by qPCR

To assess total bacterial density (copy number per millilitre (copies·mL^−1^) in sputum), qPCR of the 16S ribosomal RNA (rRNA) gene was done using a target-specific primer and probe set [12]. *P. aeruginosa* density (copies·mL^−1^) was determined using species-specific primers targeting *oprL* [13] and *ecfX* [14].

### Illumina miSeq NGS

DNA sequencing targeting the 16S rRNA marker-gene V4 region was performed using modified universal primers [15]. Raw sequence data were deposited to the European Nucleotide Archive (Study Accession: PRJEB74748). Ecological community measurements (*e.g.* relative abundance (RA) of genera, community richness, diversity, dominance, evenness and structure) were determined.

### Inflammatory biomarkers

Neutrophil elastase, interleukin-8 (IL-8), interleukin-1β (IL-1β), calprotectin and high mobility group box 1 (HMGB1) levels in sputum were determined. Blood samples were used to determine C-reactive protein (CRP) from plasma and eosinophil cell count according to standard methods.

### Observational end-points

Our primary focus was on the existence of community endotypes and their relationship with microbial, inflammatory and clinical variables. Therefore, we determined change in *P. aeruginosa* and total bacterial density (Log_10_ copies·mL^−1^; qPCR) and *Pseudomonas* (%RA; NGS) from Day 1 to Day 29 to determine the microbiological effect of TIP. Participants receiving TIP were stratified into two clusters, based on the change in *P. aeruginosa* density (*oprL* copies·mL^−1^) from Day 1 to Day 29: responders, ≥2Log_10_ reduction in density and non-responders, <2Log_10_ reduction (or an increase) in density. An arbitrary reduction of 2Log_10_ *oprL* copies·mL^−1^ was selected as this cut-off was used to inform the primary study power and sample size calculation [11]. The relationship between microbiota metrics in these endotypes and clinical and inflammatory biomarkers was also determined. Finally, we evaluated the effect of TIP (different doses and regimens) on *P. aeruginosa* density and community dynamics (Day 1 to Day 169; qPCR and NGS) [11].

### Statistical analysis

End-points (Day 1 *versus* Day 29) were analysed using a Wilcoxon signed-rank test. The correlation between different methodologies for assessing *P. aeruginosa* density and RA were compared using a Spearman's rank-correlation coefficient (ρ) with a repeated measure correlation due to multiple samples from participants.

Differences within the responder and non-responder clusters between Day 1 and Day 29 were analysed using a Wilcoxon signed-rank test. A univariable analysis was used to identify potential independent predictors for response to TIP treatment and variables with p≤0.15 were examined in a multivariable logistic regression model.

## Results

### Study population

**FIGURE 1:**
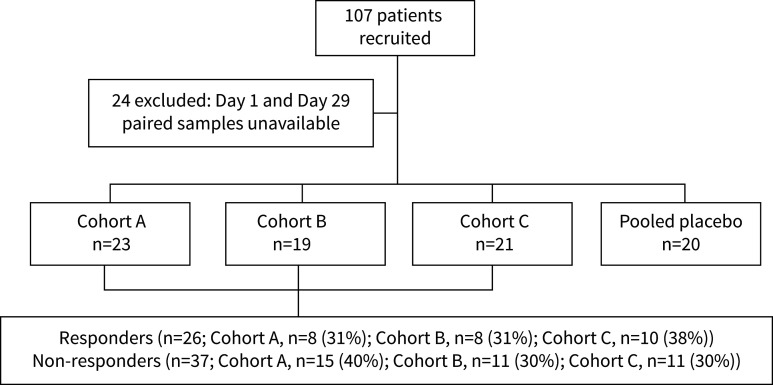
Schematic outline of iBEST-1 participants included in present study. Participants were randomised in a 1:1:1:1 ratio to one of three TIP treatment cohorts (A, 84 mg tobramycin; B, 112 mg tobramycin; C, 224 mg tobramycin) or placebo. In total 24 participants were excluded from the present analysis as they did not have available paired samples for Day 1 and Day 29, with the most common reason being that a sputum sample was not available for analysis on Day 29 (no sample available, n=8; deep throat swab collected instead, n=8; not enough sample for analysis, n=2; sample discarded due to erroneous labelling, n=2; sample was older than 48 h, n=2; missing metadata, n=2).

Most study outcomes were addressed based on 83 participants (treatment cohort: A, n=23; B, n=19; C, n=21; pooled placebo: n=20), or a subset of those, who had paired sputum samples at Day 1 and Day 29 (figure 1). The treatment and placebo cohorts were similar at Day 1 (table 1), except for *Pseudomonas* RA (p=0.007), which was lowest in cohort B. This latter finding may reflect that more samples within cohort B also had a relatively lower absolute *P. aeruginosa* abundance.

**TABLE 1:** Day 1 demographic characteristics, microbiota metrics and inflammatory biomarker profile of participants (n=83) with samples analysed in this study

		Total	Cohort A – 84 mg tobramycin	Cohort B – 140 mg tobramycin	Cohort C – 224 mg tobramycin	Pooled placebo	p-value^#^
**Participants, n**		83	23	19	21	20	
**Age years, median (IQR)**		68 (58.5–74.0)	62 (50.5–71.5)	69 (61.0–76.5)	68 (60.0–73.0)	71.5 (63.5–74.5)	0.310
**Age range years, min–max**		35–86	39–82	42–82	35–86	40–81	
**Age group, n (%)**							
≥65 years		51 (61.4)	11 (47.8)	12 (63.2)	13 (61.9)	15 (75.0)	
<65 years		32 (38.6)	12 (52.2)	7 (36.8)	8 (38.1)	5 (25.0)	0.337
**Sex, n (%)**							
Female		51 (61.4)	16 (69.6)	11 (57.9)	12 (57.1)	12 (60.0)	
Male		32 (38.6)	7 (30.4)	8 (42.1)	9 (42.9)	8 (40.0)	0.820
**FEV_1_ % predicted**							
Median (IQR)		56.9 (45.1–67.2)	52.6 (44.5–76.3)	61.4 (47.5–80.5)	52.7 (0.5–63.2)	60.1 (46.4–66.7)	0.528
Min–max		24.0–135.1	32.2–99.5	24.0–135.1	30.5–111.6	26.7–85.8	
Missing, n		1	0	0	1	0	
**FVC % predicted**							
Median (IQR)		71.5 (59.5–87.4)	67.6 (57.5–89.3)	91 (68.3–98.2)	64.9 (60.7–75.2)	71.6 (65.2–77.9)	0.093
Min–max		33.0–135.1	40.4–113.8	43.0–135.1	33.0–124.8	33.2–91.0	
Missing, n		1	0	0	1	0	
**Microbiota metrics**							
16S rRNA (Log10; copies·mL^−1^), median (IQR)		9.2 (8.8–9.6)	9.2 (8.8–9.6)	9.4 (8.9–9.8)	9.3 (8.7–9.5)	9 (8.8–9.4)	0.624
Min–max		8.0–10.3	8.0–9.9	8.0–10.1	8.2–10.3	8.4–10.0	
*oprL* (Log10; copies·mL^−1^), median (IQR)		7.9 (7.6–8.2)	8.1 (7.5–8.5)	7.9 (6.9–8.1)	7.9 (7.7–8.5)	7.9 (7.7–8.2)	0.326
Min–max		4.0–9.0	6.6–9.0	4.0–8.7	6.3–8.8	5.3–8.6	
*ecfX* (Log10; copies·mL^−1^), median (IQR)		7.9 (7.4–8.3)	8 (7.4–8.4)	7.9 (6.7–8.1)	7.9 (7.5–8.3)	7.8 (7.5–8.2)	0.582
Min–max		4.0–9.1	4.8–9.1	4.0–8.7	6.3–9.0	5.4–9.1	
*Pseudomonas aeruginosa* (Log10; CFU·g^−1^ sputum), median (IQR)		7.0 (6.1–7.9)	7.3 (6.9–8.3)	7.1 (5.9, 8.2)	6.4 (5.8–6.9)	7.1 (6.3–8.3)	0.063
Min–max		2.0–10.9	4.8–10.2	2.0–10.1	2.0–8.3	4.5–10.9	
*Pseudomonas* (% RA), median (IQR)		62.1 (24.6–88.6)	81.9 (46.8–92.6)	21.9 (3.2–60.0)	61 (36.9–86.8)	75.7 (42.8–91.9)	**0.007**
Min–max		0.0–98.8	16.2–96.6	0.0–98.8	1.5–97.1	0.4–98.4	
Shannon–Wiener Index (H), median (IQR)		1 (0.5–1.5)	0.6 (0.4–1.3)	1.2 (0.8–1.8)	1.2 (0.6–1.6)	1 (0.4–1.3)	0.201
Min–max		0.1–2.7	0.2–2.4	0.1–2.7	0.2–2.5	0.1–2.4	
Dominance (D), median (IQR)		0.6 (0.4–0.8)	0.7 (0.5–0.9)	0.4 (0.3–0.7))	0.5 (0.4–0.8)	0.6 (0.4–0.8)	0.128
Min–max		0.1–1.0	0.2–0.9	0.1–1.0	0.1–0.9	0.2–1.0	
**Inflammatory biomarkers: blood**							
CRP (mg·L^−1^), median (IQR)		8.8 (3.1–22.9)	8.9 (2.8–14.1)	8 (2.7–30.4)	8.6 (3.8–30.4)	8.4 (4.4–15.7)	0.355
Min–max		0.4–81.3	0.4–41.0	1.1–81.3	0.8–56.9	1.0–43.5	
Missing		1	0	1	0	0	
Eosinophils (×10^9^·L^−1^), median (IQR)		0.2 (0.1–0.2)	0.1 (0.1–0.2)	0.1 (0.1–0.2)	0.2 (0.1–0.2)	0.2 (0.1–0.3)	0.662
Min–max		0.0–1.7	0.0–0.4	0.0–1.7	0.0–1.1	0.0–0.7	
Missing		2	0	0	0	2	
**Inflammatory biomarkers: sputum**							
IL-8 (pg·mL^−1^), median (IQR)		11 301 (6224–18 306)	11 485 (7881–18 642)	12 199 (5013–20 195)	11 130 (4411–14 078)	11 040 (6993–20 185)	0.751
Min–max		860–44 657	860–37 −863	1152–44 657	1718–40 796	2223–35 056	
Missing		2	0	1	1	0	
Neutrophil elastase (ng·mL^−1^), median (IQR)		3670 (2568–6502)	3670 (2996–4654)	3785 (2543–6542)	3171 (2493–6723)	3469 (2568–7930)	0.807
Min–max		188–159 662	2313–47 320	2309–159 662	1289–97 873	188–123 607	
Missing		2	0	1	1	0	
IL-1β (pg·mL^−1^), median (IQR)		965 (458–2470)	1124 (677–2844)	855 (524–2291)	908 (328–2094)	1024 (550–2830)	0.656
Min–max		36–10 345	125–5235	83–3051	36–10 345.	106–7482	
Missing		2	0	1	1	0	
HMGB1 (ng·mL^−1^), median (IQR)		0.4 (0.4–2.8)	0.4 (0.4–1.3)	0.6 (0.4–5.2)	0.4 (0.4–13)	0.4 (0.4–2.6)	0.611
Min–max		0.4–191.8	0.4–89.0	0.4–88.5	0.4–93.0	0.4–191.8	
Missing		2	0	1	1	0	
Calprotectin (ng·mL^−1^), median (IQR)		25 668 (10 783–42 655)	29 310 (16 971–43 165)	28 574 (9385–36 100)	19 822 (9171–48 466)	21 734 (12 370–39 235)	0.995
Min–max		82–118 720	82–79 473	541–101 802	1939–90 927	4135–118 720	
Missing		2	0	1	1	0	

FEV_1_: forced expiratory volume in 1 s; FVC: forced vital capacity; CFU: colony-forming units: RA: relative abundance; CRP: C-reactive protein; IL: interleukin; HMGB1: high-mobility group box 1 protein. ^#^: statistical comparisons made between cohort A, cohort B, cohort C and the pooled placebo. Kruskal–Wallis test; p<0.05 denotes statistical significance.

### Change in bacterial density (Day 1 to Day 29)

**FIGURE 2:**
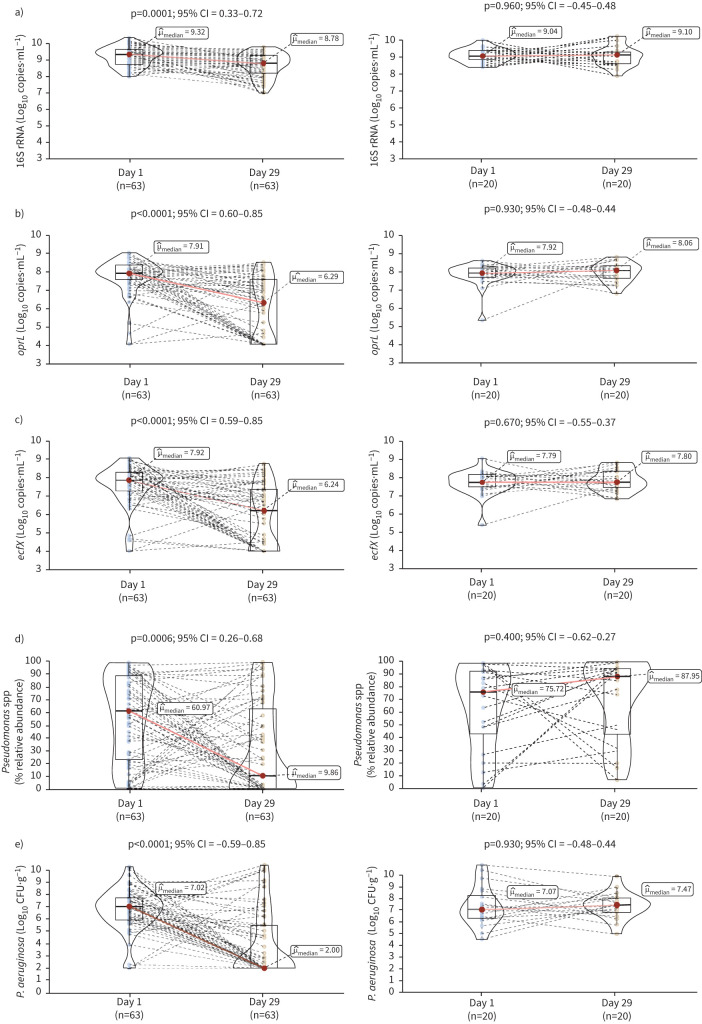
Comparison between Day 1 and Day 29 for participants in the TIP treatment (left-hand side) and pooled placebo (right-hand side) cohorts. a) Total bacterial density (16S rRNA copies·mL^−1^), b) *Pseudomonas aeruginosa* density (*oprL* copies·mL^−1^), c) *P. aeruginosa* density (*ecfX* copies·mL^−1^), d) *Pseudomonas* spp. (% relative abundance) and e) *P. aeruginosa* density (CFU·g^−1^ sputum). p<0.05 denotes statistical significance.

A significant reduction in total bacterial (figure 2a) and *P. aeruginosa* density (*oprL* (figure 2b) and *ecfX* (figure 2c) copies·mL^−1^) between Day 1 and Day 29 was observed for participants in the TIP treatment (n=63; p≤0.0001) but not placebo (n=20; p>0.05) group. Similar trends were observed for *Pseudomonas* RA from NGS (TIP, p=0.0006; placebo, p=0.400) (figure 2d) and for *P. aeruginosa* density determined previously [7] by quantitative culture (TIP, p<0.0001; placebo, p=0.930) (figure 2e). Furthermore, the highest dose of TIP (cohort C) demonstrated the most marked reduction in *P. aeruginosa* density/*Pseudomonas* RA by all methods (supplementary figure 1).

There was a strong positive correlation between the two *P. aeruginosa*-specific qPCR assays (ρ=0.931; p<0.0001); therefore, data from one assay (*oprL*) was selected for further analyses. Similarly, significant positive correlations were apparent between *P. aeruginosa* density measured by qPCR and *Pseudomonas* RA by NGS (ρ=0.890; p<0.0001; supplementary figure 2A) and culture (ρ=0.720; p<0.0001; supplementary figure 2B). *Pseudomonas* RA and *P. aeruginosa* density by culture (ρ=0.540; p<0.0001; supplementary figure 2C) also correlated strongly.

### Change in *P. aeruginosa* density with treatment

In the TIP treatment group, two clusters were observed based on microbiological response (figure 2); 26 participants were stratified as responders (≥2Log_10_ reduction in *P. aeruginosa* density) and 37 as non-responders (<2Log_10_ reduction in *P. aeruginosa* density). Day 1 characteristics of responders and non-responders are shown in supplementary table 1. There was no association between the dose of TIP (*i.e.* cohort) and cluster (χ^2^=0.754; p=0.686).

A slight reduction (<1Log_10_) in total bacterial density was observed in both the responder ($\tilde{\mu }$_median_=9.22 *versus* $\tilde{\mu }$_median_=8.74; p=0.020) and non-responder ($\tilde{\mu }$_median_=9.35 *versus* $\tilde{\mu }$_median_=8.88; p<0.0001) clusters (supplementary figure 3A). *P. aeruginosa* density decreased in the responder cluster by ∼4Log_10_ ($\tilde{\mu }$_median_=7.94 *versus* $\tilde{\mu }$_median_=4.00; p<0.0001) compared to <1Log_10_ in the non-responder cluster ($\tilde{\mu }$_median_=7.90 *versus* $\tilde{\mu }$_median_=7.25; p=0.090) (supplementary figure 3B). In the responder cluster, no *P. aeruginosa* was detected by qPCR (*i.e.* density<limit of detection) at Day 29 in 20 out of 26 samples compared with three out of 37 in the non-responder cluster. Similar trends were observed for *Pseudomonas* RA (NGS) and *P. aeruginosa* density by culture (Log_10_ CFU·g^−1^) (supplementary figure 3C–D). All participants in the responder cluster and 32 out of 37 participants in the non-responder cluster had a Day 1 *P. aeruginosa* isolate that was tobramycin susceptible. At Day 29, only three participants in the responder cluster were culture-positive for *P. aeruginosa* with one demonstrating a change in tobramycin susceptibility category (susceptible to intermediate). Of the 24 out of 37 participants who were culture-positive for *P. aeruginosa* at Day 29 in the non-responder cluster, only four had a change in tobramycin susceptibility category (susceptible/intermediate to resistant).

**FIGURE 3:**
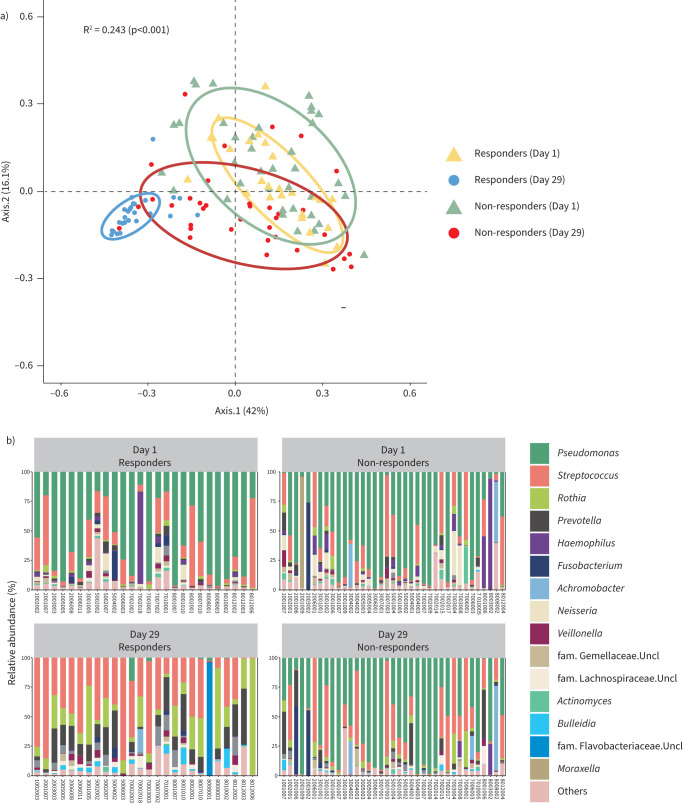
a) Principal coordinates analysis (PCoA) plot comparing microbial communities in TIP treatment response groups (responders, ≥2Log_10_ reduction in *P. aeruginosa* density; non-responders, <2Log_10_ reduction in *P. aeruginosa* density) based on Hellinger transformed genus-level taxonomic composition and Bray–Curtis dissimilarity metrics separated by visit day (Day 1 and Day 29). Comparisons are based on PERMANOVA analysis (permutational multivariate ANOVA) as implemented within the ADONIS function of the vegan-package in R. p<0.05 denotes statistical significance and confidence (ellipses) is based on 90% CI. b) Relative abundance (%) of the top 15 taxa in paired samples at Day 1 and Day 29 by treatment response.

There was also a significant shift in community structure (β-diversity) from Day 1 to Day 29 in the responder cluster with clear separation between time-points (yellow and blue ellipses; figure 3a). In contrast, in the non-responder cluster, only a moderate shift in community structure was observed (green and red ellipses overlap; figure 3a). Furthermore, changes in community composition in individual participants were more apparent for responders than non-responders (figure 3b). In the responder cluster, there was a reduction in the RA of *Pseudomonas*, *Haemophilus* and *Neisseria* (p<0.0001) and an increase in the RA of *Bulleidia*, *Porphyromonas*, *Prevotella*, *Rothia* and *Streptococcus* (p<0.0001 to p=0.002) (supplementary figure 4A). In comparison, in the non-responder cluster, there was a reduction in the RA of *Haemophilus* and *Neisseria* (p<0.0001) and an increase in *Rothia* (p=0.027) RA but no change in the RA of any other taxa including *Pseudomonas* (supplementary figure 4B). A reduction in community dominance (p=0.018) and increased evenness (p=0.006) were observed in the responder cluster (supplementary figure 4A), while decreased richness (p=0.009) was apparent in the non-responder cluster (supplementary figure 4B).

**FIGURE 4:**
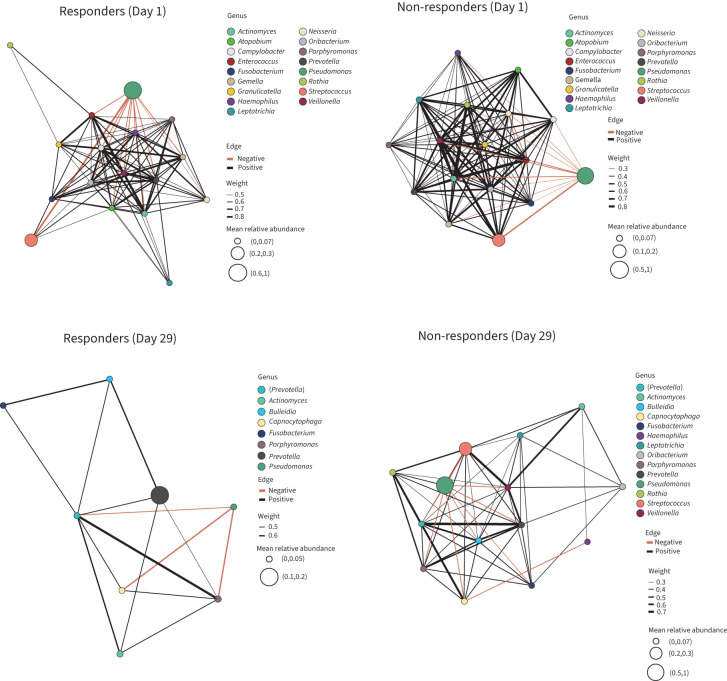
Taxa co-occurrence networks at Day 1 and Day 29 in TIP treatment response groups (responders, ≥2Log_10_ reduction in *Pseudomonas aeruginosa* density; non-responders, <2Log_10_ reduction in *P. aeruginosa* density). Co-occurrence networks are based on taxa observed in over 65% of all samples with valid association threshold ≤−0.3 (negative) and ≥0.3 (positive) for a significant correlation identified with a p<0.05 following Benjamini–Hochberg adjustment for multiple testing.

Taxa co-occurrence network analysis revealed that complex interactions were apparent at Day 1 in both clusters with several obligate, *e.g.*, Prevotella, Veillonella, and facultative, e.g., Streptococcus and Rothia, anaerobes forming the main connected component of the networks. *Pseudomonas* demonstrated a negative correlation (mutual exclusion) with other community members (figure 4). By Day 29, there was clear disruption of the network and putative connections between taxa in the responder cluster, with most of the main Day 1 members absent (reduction from 17 to 8 members). *Pseudomonas* remained a network member at Day 29 but was present in a diminished proportion (figure 4). In contrast, simplification of the co-occurrence network was less evident in the non-responder cluster (reduction from 17 to 14 members) and the main Day 1 community members (*e.g. Pseudomonas*, *Streptococcus*) still contributed to the network in similar proportions. An Analysis of Composition of Microbiomes with Bias Correction (ANCOMBC) confirmed these observations for the main taxa (details provided in supplementary material including supplementary table 2).

Following treatment, a significant (p<0.05) decrease in neutrophil elastase and IL-1β and an increase in HMGB1 were observed in both clusters. Additional significant changes were observed in the responder cluster only with CRP (p=0.014), IL-8 (p=0.0003) and calprotectin (p=0.048) decreasing and eosinophil count increasing (p=0.018) (supplementary figure 5A–G). Although not statistically significant (p=0.117), fewer participants in the responder cluster (seven out of 26; 27%) experienced an exacerbation by the end of the trial period (Day 169) than in the non-responder cluster (18 out of 37; 49%).

Lastly, lung function was higher at Day 1 in responders ($\tilde{\mu }$_median_=64.6% predicted) compared to non-responders ($\tilde{\mu }$_median_=50.3% predicted) (odds ratio(OR) 1.035, 95% confidence interval (CI) 1.009–1.064; p=0.013). The number of exacerbations in the previous 12 months was also higher in the responder ($\tilde{\mu }$_median_=2.5) compared to the non-responder ($\tilde{\mu }$_median_=2.0) cluster (OR 1.578, 95% CI 0.987–2.524; p=0.057). After controlling for these variables in a multivariable logistic regression, only lung function independently predicted response to TIP treatment (supplementary table 3). There was no difference between clusters at Day 1 with respect to age, sex, or total bacterial or *P. aeruginosa* density. Likewise, there was no difference in the main microbiota metrics or inflammatory biomarkers (supplementary table 3).

### Microbiota community trajectory

Although TIP treatment resulted in a reduction in *P. aeruginosa* density (*oprL* copies·mL^−1^) for the first 28 days (all doses/regimens), there was a noticeable increase in *P. aeruginosa* density during periods when TIP was not prescribed (cyclical treatment) for cohorts B and C (supplementary figure 6A/B). Similar findings were observed when principal coordinate plots were compared (supplementary figure 6C). At Day 1, there was no significant separation between cohorts (p=0.351). However, TIP treatment was associated with a clear separation between cohorts which was maintained until treatment stopped at Day 113 (p<0.05). In the cyclical regimen, there was a notable shift towards the pooled placebo group when the treatment cohorts received placebo. During the follow-up period, there was no significant separation between cohorts (Day 141, p=0.185; Day 169, p=0.392).

## Discussion

We report, to our knowledge, the first clinical study in patients with BE in which molecular diagnostic techniques were used to measure microbiological efficacy end-points and stratify patients into endotypes which predicted response to antimicrobial treatment. There was a significant reduction in total bacterial and *P. aeruginosa* density, measured by qPCR, between Day 1 and Day 29 in the TIP treatment but not placebo group. These findings are consistent with results previously published for *P. aeruginosa* density by quantitative culture [7] with strong positive correlations apparent between the methods. Bacterial density is a key biomarker of disease severity, exacerbation frequency and morbidity in patients with BE and predicts response to inhaled antibiotics in terms of respiratory symptoms [4, 16, 17]. Our findings suggest that qPCR, which is less time-consuming and labour intensive than quantitative culture, could potentially be used as a surrogate end-point to measure treatment effect in clinical trials.

In BE, clinical trials targeting chronic bacterial infection with antibiotics have repeatedly failed to reach their end-points or showed benefit in only a subset of patients [18–20]. It was recently proposed that this response heterogeneity is due to the existence of inflammatory molecular endotypes [8]; four molecular endotypes were associated with distinct microbiome profiles and future exacerbation risk with lower diversity associated with more severe inflammation. Although the underlying mechanisms are unclear, within the TIP treatment group, we identified two BE community endotypes, differentiated by response to TIP targeting *P. aeruginosa*, which could not be explained by the dose received. At Day 1, the microbiota profiles of the two endotypes were similar; however, at Day 29, there was a significant shift in microbiota profile in the responder cluster which was not as apparent in the non-responder cluster. This shift was primarily driven by a reduction in the abundance of known pathogens and an increase in taxa considered commensal bacteria. The elimination of many inter-taxa connections in the core community of the responder endotype also indicated that, in addition to the direct effect on *P. aeruginosa,* there was an off-target effect following TIP treatment. Importantly, the reduction in *Pseudomonas* dominance in the responder endotype was associated with decreased inflammation and fewer patients experiencing exacerbations in the following months. This confirms the findings of previous studies which showed that PEx dynamics were driven by pathogen dominance (*e.g. P. aeruginosa*) [21] and that *Pseudomonas*-dominated microbiome profiles were associated with exacerbation frequency, hospitalisation risk and mortality [8, 22].

Most participants had a Day 1 *P. aeruginosa* isolate that was susceptible to tobramycin, and there was no trend towards an increase in resistance. This finding agrees with previous studies which reported that long-term inhaled antibiotic treatment in BE only occasionally results in resistance development [23–25]. Therefore, this microbiological phenotype does not appear to explain the differences in response to TIP. Although susceptibility testing remains an important assessment, lack of agreement between *in vitro* antimicrobial susceptibility of individual pathogens and clinical outcomes is recognised in BE. Furthermore, a recent study, characterising the resistome in patients with BE revealed two resistotypes, each demonstrating a distinct resistance pattern and patient clinical characteristics [10]. Targeted *P. aeruginosa* eradication resulted in a shift to a resistotype associated with reduced multidrug resistance and resistance gene diversity. It is possible that in our cohort, different resistome profiles were present at Day 1 that associate with response to TIP treatment.

When we compared Day 1 demographic, microbiota and inflammatory biomarker characteristics, lung function was higher in the responder endotype and independently predicted a response to TIP treatment. As tobramycin concentration in sputum was not measured, we could not determine whether this difference in lung function between the two clusters was associated with a difference in TIP penetration into the airways. Current guidelines recommend long-term inhaled antibiotics in patients with BE who experience ≥3 exacerbations/year [5]. However, our findings suggest that in individuals with chronic *P. aeruginosa* infection, early intervention with inhaled antibiotics such as TIP should be considered when lung function is higher; this is more likely to result in a significant reduction in *P. aeruginosa* density in the airways and an associated reduction in exacerbation frequency, morbidity and mortality [4, 8, 16].

We also compared the effect of continuous *versus* cyclical TIP treatment on the airway microbiota. As noted in previous studies [18, 20], off-treatment periods were associated with an increase in *P. aeruginosa* density towards baseline values. Furthermore, off-periods have been associated with a relapse in symptoms, suggesting that continuous bacterial suppression may be beneficial for symptom control [17]. However, it may also result in a less diverse community over time, which is associated with worse long-term clinical outcomes [8, 22, 26].

The study has several limitations. First, the sample size and the fact that only *P. aeruginosa* positive patients were recruited limits the generalisability of the findings to the broader BE population, and the subgroup analyses could potentially introduce bias. Second, stratifying patients into responders and non-responder endotypes based on the change in *P. aeruginosa* density by qPCR could introduce subjectivity and may not capture the full complexity of treatment response. Nevertheless, the cut-off selected for stratification was based on the primary study protocol that deemed a reduction in *P. aeruginosa* density of ≥2Log_10_ to be a clinically meaningful response to TIP treatment. Treatment effects of inhaled antibiotics over 28 days on *P. aeruginosa* density are on average around this cut-off value in clinical trials [7, 21, 27, 28]. Furthermore, we also performed a sensitivity analysis using alternate reductions in *P. aeruginosa* density (1Log_10_ and 3Log_10_ *oprL* copies·mL^−1^) for stratifying participants as responders and non-responders, and the same trends in the microbiota metrics were evident (supplementary material). Third, the endotypes identified require further characterisation including patient radiological, genetic and other immunological factors not available here. In addition, the findings observed in this study with 28 days of TIP treatment may not capture long-term microbial or clinical outcomes. Therefore, future trials are needed to both validate the existence of the endotypes identified in an independent cohort and to follow-up the responders and non-responders over an extended period. Fourth, our DNA extraction method did not involve depletion of human DNA; therefore, shotgun metagenomic sequencing could not be performed to determine whether there were differences in the resistome in responder and non-responders. Further trials could include shotgun-metagenomic sequencing or metatranscriptomic analysis to assess functional changes in the microbiota including to provide insights into antimicrobial resistance dynamics. Finally, we did not exclude DNA from non-viable bacteria from our samples; however, qPCR and culture results strongly correlated.

In BE, the heterogeneous response to antimicrobial treatment underscores the need for precision medicine approaches that account for underlying microbial and inflammatory profiles. By defining endotypes based on treatment response, this study provides a framework for personalised therapy, offering insights into microbial–immune interactions and identifying putative predictors of clinical outcomes to optimise patient care.

## Supplementary material

**Please note:** supplementary material is not edited by the Editorial Office, and is uploaded as it has been supplied by the author. Supplementary material 00389-2025.SUPPLEMENT Supplementary material 00389-2025.SUPPLEMENT Figure S1 00389-2025.SUPPLEMENT Figure S2 00389-2025.SUPPLEMENT2 Figure S3 00389-2025.SUPPLEMENT3 Figure S4 00389-2025.SUPPLEMENT4 Figure S5 00389-2025.SUPPLEMENT5 Figure S6 00389-2025.SUPPLEMENT6
