# Supplementary material for: Molecular endotyping in people with bronchiectasis based on response to antibiotic treatment: iBEST study
Source: ERJ Open Res. 2025 Dec 29;11(6):00389-2025. doi: 10.1183/23120541.00389-2025 (PMC12746121; doi:10.1183/23120541.00389-2025)
Supplement: Supplementary file 2 [file 00389-2025.SUPPLEMENT.pdf]

Online Supplement

Molecular endotyping in people with bronchiectasis based on response to antibiotic treatment: iBEST study

Gisli G. Einarsson[1, 3,*], Laura J. Sherrard[1, 3,*], Andrew J. Lee[1, 3], Jack Carson[1, 3], Andrew Marshall[1, 3], Aya Alkhatib[1, 3], Vanessa Brown[1, 2], Deirdre F. Gilpin[1, 3], Gerhild Angyalosi[4], Michael R. Loebinger[5, 6], James D. Chalmers[7], Francesco Blasi[8, 9], Charles S. Haworth[10, 11], Eva Polverino[12], Harm A.W.M. Tiddens[13, 14], Herman Goossens[15], Felix C. Ringshausen[16], Adam T. Hill[17], J. Stuart Elborn[1, 2,‡], Michael M. Tunney[1, 3,‡]

[1] Queen's University Belfast, Belfast, UK.

[2] Centre for Experimental Medicine, Queen's University Belfast, Belfast, UK.

[3] Halo Research Group, School of Pharmacy, Queen's University Belfast, Belfast, UK.

[4] Novartis Pharma AG, Basel, Switzerland.

[5] Host Defence Unit, Royal Brompton Hospital and Harefield NHS Foundation Trust, London, UK.

[6] Imperial College London, London, UK

[7] Scottish Centre for Respiratory Research, University of Dundee, Ninewells Hospital and Medical School, Dundee, Scotland, UK.

[8] Internal Medicine Department, Respiratory Unit and Adult Cystic Fibrosis Center, Fondazione IRCCS Cà Granda Ospedale Maggiore Policlinico, Milan, Italy.

[9] Department of Pathophysiology and Transplantation, Università degli Studi di Milano, Milan, Italy.

[10] Cambridge Centre for Lung Infection, Royal Papworth Hospital NHS Foundation Trust, Cambridge, UK.

[11] Department of Medicine, University of Cambridge, Cambridge, UK.

[12] Respiratory Disease Department, Vall d' Hebron University Hospital –VHIR, Barcelona, Spain.

[13] Dept of Paediatric Pulmonology and Allergology, Erasmus Medical Centre Sophia Children's Hospital, Rotterdam, The Netherlands.

[14] Dept of Radiology and Nuclear Medicine, Erasmus Medical Centre, Rotterdam, The Netherlands.

[15] Dept of Clinical Microbiology, University Hospital Antwerp, Antwerp, Belgium.

[16] Dept of Respiratory Medicine, Hannover Medical School, and Biomedical Research in End-stage and Obstructive Lung Disease Hannover (BREATH), German Center for Lung Research (DZL), Hannover, Germany.

[17] Dept of Respiratory Medicine, Royal Infirmary of Edinburgh, and University of Edinburgh, Edinburgh, UK.

* GGE and LJS are joint first authors on this paper

‡ JSE and MMT are joint senior authors on this paper

Corresponding author: Michael M. Tunney, Queen's University Belfast, Belfast, United Kingdom. Email: m.tunney@qub.ac.uk

Additional methodological details

Participants and sample collection

Sputum samples were stored at -80°C until processing. Blood samples were also collected for inflammatory biomarker analyses.

Participant characteristics recorded included gender, age, spirometry measurements, and occurrence of pulmonary exacerbations in the 12-months prior to the first study visit and during the clinical trial. Most pulmonary exacerbations were mild to moderate, with only five patients (4.7%) experiencing severe episodes. No deaths were reported. More detailed information related to adverse events has previously been described by Loebinger et al. [1].

Pseudomonas aeruginosa characteristics determined previously and used in the present study included density by culture (colony forming units per gram of sputum) and tobramycin minimum inhibitory concentration [1]. In the primary study, all *P. aeruginosa* isolates were tested for antibiotic susceptibility including to tobramycin, by broth microdilution following the recommended methodology of the CLSI [CLSI M07-A10E], with results reported according to the EUCAST guidelines.

Determination of total bacterial and P. aeruginosa density by qPCR

To assess total bacterial density (copy number per millilitre [copies/mL] in sputum), quantitative polymerase chain reaction (qPCR) of the bacterial 16S ribosomal RNA (rRNA) gene was done using a target specific primer and probe set as previously described [2]. In addition, *P. aeruginosa* density (copies/mL) was determined using species specific primers targeting either *oprL* [3] or *ecfX* [4], as previously described. Two targets were initially used to clarify if one gene was more informative that the other; however, there was a strong agreement between both targets and only one instance of an obvious discrepancy. Assays were performed

in triplicate with positive, negative, and non-template controls. The lower limit of detection (LOD) was determined as $1x10^4$ gene copies/mL to account for the relevant dilution steps used during the qPCR assay. The number of *P. aeruginosa* specific-gene copies was normalised to total bacterial density based on the approximation that *P. aeruginosa* strains contain an average of four copies of the 16S rRNA operon in their genome.

Generation and processing of 16S rRNA amplicon sequences for microbiota analysis

Characterisation of airway microbiota composition and structure was determined by DNA sequencing targeting the V4 region of the 16S rRNA marker-gene in a two-step library preparation, applying modified universal primers as previously described [5].

Sample pre-lysis and DNA extraction from sputum samples

All sputum samples underwent a pre-lysis step before DNA extraction using the Roche MagNA Pure 96 System (Roche Diagnostics Ltd., UK). To prepare the 10% Sputolysin solution, 1.8 mL of sterile nuclease-free water was added to a pre-aliquoted Sputolysin stock. If the sputum samples were frozen, they were thawed on ice and then placed in a sterile Petri dish. Plugs were separated from any saliva-like material using scalpels or sterile microbiological loops, and a 100 mg (+/- 20 mg) sputum plug was selected and placed in a sterile Eppendorf tube.

Subsequently, 100 µL of the 10% Sputolysin solution was added to the sputum plug, followed by vortex mixing. The mixture was then incubated at room temperature for 30 minutes in a heated shaker set at 2000 rpm. If necessary, the sample was further homogenized with pipette mixing. A working solution of lysozyme was prepared by dissolving an aliquot of lysozyme in Roche Bacteria Lysis Buffer (BLB) to achieve a concentration of 5 mg/mL. A 200

µL aliquot of this lysozyme solution was added to each sample, which was then vortex mixed and incubated at 37°C for 30 minutes in a heated shaker at 2000 rpm.

The samples were then transferred to glass bead tubes and agitated in a Fast-Prep rotor stator homogenizer at speed 6.0 for 40 seconds, ensuring that the tube lids were tightly fastened. Following this, the samples were centrifuged at 13,000 x g for 1 minute. Next, 32 µL of proteinase K (from a Qiagen stock at 20 mg/mL) was added to each sample, which was vortex mixed and incubated at 65°C for 10 minutes in a heated shaker set at 1500 rpm.

To continue the process, 150 µL of nuclease-free water was added to the samples, which were then agitated again in the Fast-Prep homogenizer at speed 6.0 for 40 seconds, with lids tightly fastened. The samples were then incubated at 95°C for 10 minutes in a heated shaker at 1000 rpm. Following incubation, the samples were centrifuged at 10,000 x g for 10 minutes at 4°C. Finally, 200 µL of the supernatant was carefully transferred to a fresh 1.5 mL Eppendorf tube, avoiding any debris, and stored at -80°C until further use.

Polymerase chain reactions (PCRs)

In preparation for Illumina MiSeq sequencing of the 16S rRNA marker-gene, the following steps were performed:

PCR 1 This step involved pre-amplification of the 16S rRNA marker gene region. Here, the PCR was performed using ~200 ng of gDNA from each sample. A mastermix solution was prepared using non-modified primers targeting positions 515F and 806R within the V4 region [5 µL 5x Phusion Hifi Buffer, 0.5 µL (10 mM) dNTP, 1 µL (10µM) V4 primer mix; 0.25 µL Phusion HS II polymerase and diethylpyrocarbonate [DEPC]-treated water to 25 µL per reaction] and amplification was done using the following conditions: 98°C for 30 seconds (x1) →98°C for 10 seconds + 52°C for 30 seconds + 72°C for 20 seconds (10 cycles) →72°C for 5 minutes →hold at 4°C for ∞. Thereafter, the PCR products were cleaned using a AxyPrep Mag

PCR Clean-up kit. Briefly, after vortexing the magnetic beads, 15 μL of the Axygen beads was added to 10 μL of PCR product in a sterile 96-well plate, mixed well and incubated at room temperature for 5 minutes. The reaction plate was placed onto a Axygen IMAG Magnetic Beads Separation Device until the liquid turned clear: this clear liquid was removed from the plate and discarded. Next, 180 μL of 70% ethanol was added to each well and incubated for 30 seconds at room temperature, and then the ethanol was removed and discarded. The previous step was repeated once. The beads were allowed to air dry for no more than 5 minutes. Nuclease free water (11 μL) was added to each well and the reaction plate was removed from the Separation Device and the contents of the wells were mixed well by gentle vortexing. The reaction plate was returned to the Separation Device for 1 minute to separate the beads from the solution. Finally, 10 μL of the cleaned-up PCR 1 product was transferred to a sterile 96-well plate for the next PCR step.

PCR 2 This step involved Reverse Tagging using the cleaned product from PCR 1 and an equimolar mixture of the reverse frame-shift (FS) primers 808R_f1, 808R_f2, 808R_f3, 808R_f4, 808R_f5, 808R_f6. Primers were combined into a working stock of 0.5 μM. A 1 cycle PCR was performed using 10 μL of the product from PCR 1. To do this, a mastermix solution [5 μL 5x Phusion Hifi Buffer, 0.5 μL (10 mM) dNTP, 2 μL (0.5 μM) Reverse_MT_tag Primer mix; 0.25 μL Phusion HS II polymerase and 7.25 μL DEPC-treated water] was prepared and amplification was achieved using the following conditions: 98°C for 60 seconds (x1) →98°C for 10 seconds + 50°C for 30 seconds + 72°C for 60 seconds (1 cycle) → hold at 4°C for ∞. Next, the PCR products were cleaned using the AxyPrep Mag PCR Clean-up kit as described above. The cleaned-up PCR 2 product (10 μL) was transferred to a sterile 96-well plate for the next PCR step.

PCR 3 This step involved Forward-Tagging using the cleaned product from PCR 2 and an equimolar mixture of the forward frame-shift (FS) primers 515F_f1, 515F_f2, 515F_f3, 515F_f4, 515F_f5, 515F_f6. Primers were combined into a working stock of 0.5 µM. A 1 cycle PCR was performed precisely as described for PCR 2 except that the Forward_MT_tag Primer mix was used with 10 µL of product from PCR 2. The PCR products from PCR 3 were then cleaned using the AxyPrep Mag PCR Clean-up kit as described above except that 17.5 µL of Axygen beads was initially added to 10 µL of PCR product in the sterile 96-well plate and after the ethanol/air-drying step, 16 µL of DEPC-treated water was added to each well. Finally, 15 µL of the cleaned-up PCR 3 product was transferred to a sterile 96-well plate for the next PCR step.

PCR 4 This step involved Nextera-Adapter/Indexing Amplification by performing a 34 cycle PCR, targeting the V4 region of the 16S rRNA marker gene, using 15 µL of the cleaned reverse and forward tagged product from the PCR 3 step. Each reaction had the same forward primers and a unique reverse primer which acted as the index (barcode) for each sample. The forward and reverse primers were typically diluted to a working stock of 5 µM. A mastermix was prepared [10 µL 5x Phusion Hifi Buffer, 1 µL (10 mM) dNTP, 2.5 µL forward primer (SEQ_V4_F; AATGATACGGCGACCACCGAGATCTACACGCCTCCCTCGCGCCATCAGAGATGTG); 2.5 µL reverse primer (INDEX_R_bc1 to bc96; CAAGCAGAAGACGGCATACGAGAT XXXXXXXX GTGACTGGAGTTCAGACGTGTGCTC); 0.5 µL Phusion HS II polymerase and 7.25 µL DEPC-treated water] and amplification was done using the following conditions: 98°C for 30 seconds (x1) →98°C for 10 seconds + 63°C for 30 seconds + 72°C for 30 seconds (34 cycle) →hold at 4°C for ∞. Thereafter, 5 µL of each reaction was run on a 1% agarose gel to confirm visually the presence of desired products (~453 bp). The PCR products from PCR 4

were then cleaned-up using the AxyPrep Mag PCR Clean-up kit as described above except that 35 µL of Axygen beads was initially added to the entire PCR product in the sterile 96-well plate and after the ethanol/air-drying step, 50 µL of DEPC-treated water was added to each well. The entire volume of the cleaned-up PCR product was transferred to a sterile 96-well plate. The products were quantified using a Quant-iT™ PicoGreen® dsDNA Assay kit (Life Technologies, UK) in a 96-well plate using 2 µL of cleaned product and according to the manufacturer's instructions. Next, equimolar amounts from each sample were pooled, adding no more than 20 µL of each reaction to the final pool. The pool was purified by running it on a 1% agarose gel, followed by excising the correct size band (~453bp) using the QIAEX II kit (Qiagen, UK) according to the manufacturer's instructions. The final pooled sample was quantified in triplicate using the Quant-iT™ PicoGreen® dsDNA Assay kit (Life Technologies, UK) according to the manufacturer's instructions. Samples were stored at -20°C/-80°C until submission for Illumina MiSeq 16S rRNA marker-gene sequencing.

Culture-independent analysis

Samples were joined together and de-multiplexed according to unique barcode sequences using the QIIME 1.9.1 pipeline [6]. The PhiX internal sequencing control was removed by aligning all sequences against the PhiX genome [7] using the bbduk.sh shell script from the BBTools package (available at https://jgi.doe.gov/data-and-tools/bbtools) where unaligned reads were retained. Sequences were clustered into their representative operational taxonomic units (OTUs) at 97% sequence identity using the UCLUST algorithm [8] in a *de novo* reference style. A representative sequence from each OTU was chosen based upon abundance within that OTU and taxonomy was assigned using the RDP naïve Bayesian classifier [9] against the QIIME compatible Greengenes 13.8 database.[10, 11] The resulting OTU table (.biom) was converted to a tab-spaced text file to assess the prevalence of OTUs within sputum samples (n=610),

positive-controls (n=4 per sequencing run) and negative-controls (n=4 per sequencing run), which were included throughout both DNA extraction and library preparation procedures.

OTUs that occurred in the background of the negative controls were compared to those observed in the sputum samples and any OTUs considered contaminants were filtered from the dataset prior to further analysis. Several OTUs were detected in the background of the negative controls, and OTUs that accounted for >90% (OTUs >0.5%) of the reads in the four negative controls had a low contribution in the sputum samples accounting for 0-0.008% of the total read number. Within the negative controls the most abundant OTUs belonged to taxa such as members of family *Halomonas* spp., *Shewanella* spp., Comamonadaceae Unclassified, family Oxalobacteraceae Unclassified, family Methylophilaceae Unclassified, *Dechloromonas* spp., *Ralstonia* spp., *Sediminibacterium* spp., family Bradyrhizobiaceae Unclassified, order and Elusimicrobiales Unclassified.

Furthermore, OTUs representing potential human sequences, Archaea, Cyanobacteria and unassigned OTUs were filtered out and treated as contaminating sequences prior to all downstream analysis.

Inflammatory biomarkers

Quantification of inflammatory biomarkers in sputum was performed for the following targets: neutrophil elastase (in house-assay that used soluble sputum supernatant) [12], interleukin 8 (IL8) (Simple Plex Human IL-8 beta ran on the ELLA platform; Bio-Techne Ltd.), interleukin 1β (IL1β) (Simple Plex Human IL-1β ran on the ELLA platform), calprotectin (Calprotectin ELISA FineTest, Wuhan Fine Biotech Co., Ltd.) and high mobility protein 1 (HMGB1) (HMGB1 ELISA IBL; Tecan). Blood samples were used to determine C-reactive protein (CRP) in plasma (CRP, Meso Scale Diagnostics, USA) and eosinophil cell count using automated analysers (haematology services).

Further details of statistical analyses and ecological community measurements

Continuous variables are reported as medians, interquartile range (IQR) and range (Min-Max) and categorical variables as counts and percentage (%). Non-parametric data was Log_{10} transformed as appropriate.

Categorical data was analysed using a Pearson's chi-squared test or Fisher's exact test, as appropriate. Participant characteristics (continuous variables) between groups (treatment cohorts and placebo) were compared using a Kruskal-Wallis test with a Dunn's test used for post-hoc analysis between cohorts, when appropriate.

A single R object was created from the .biom formatted OTU table, containing representative sequences and associated clinical metadata using phyloseq [13] for subsequent analysis in R. Calculations of microbiota/ecological indices was performed using PAST4 (https://folk.uio.no/ohammer/past/). All statistical analyses were performed in R (ver. 4.1.2) and RStudio (ver. 2021.09.2) using the packages phyloseq (ver. 1.38.0) [13], microbiome (ver. 1.19.1) [14], MicrobiotaProcess (ver. 1.6.3) [15], vegan (ver. 2.5.7) [16], dplyr (ver. 1.1.2) [17], ggplot2 (ver. 3.4.2) [18], ggpubr (ver. 0.6.0) [19], ggstatsplot (ver. 0.9.1) [20], ggtext (ver. 0.1.1) [21], Hmisc (ver. 4.6.0) [22], reshape (ver. 0.8.8) [23] and rmcorr (ver. 0.4.5) [24]. All samples were randomly sub-sampled to 11,000 reads for diversity and dissimilarity calculations.

Alpha-diversity (within group) indices calculated, such as community richness (S), diversity (Shannon-Wiener index; H'), evenness ($e^{H/S}$) and dominance (D) were compared between time-points (Day 1 vs. Day 29) in the responder and non-responder clusters using the Wilcoxon signed-rank test (2 groups). Beta-diversity (between groups) was assessed using distance-based metrics (Bray Curtis distance) on Hellinger transformed count data and

presented as a principle coordinates plot (PCoA) showing variance explained for the first two components. Furthermore, co-occurrence network analysis was performed between Day 1 and Day 29 in both responder and non-responderclusters using SpiecEasi estimation (Sparse InversE Covariance estimation for Ecological Association and Statistical Inference). Additional investigations of differences between taxa were performed using an ANCOMBC (Analysis of Composition of Microbiomes with Bias Correction) analysis.

A univariable analysis was used to identify potential independent predictors for response to TIP treatment and variables with $p \leq 0.15$ were examined in a multivariable logistic regression model using backwards stepwise selection. Co-variates were assessed for collinearity prior to their inclusion in the analysis. Age and gender were retained to control for any potential confounding effects. Adjusted odds and 95% confidence intervals are reported.

A sensitivity analysis was also performed using alternative reductions in *P. aeruginosa* density ($1Log_{10}$ and $3Log_{10}$ *oprL* copies/mL) for stratifying participants as responders and non-responders.

For longitudinal comparisons canonical ordination plots (CAP) were created using Hellinger transformed OTU count data based on visit time-point and treatment cohort (dosing: continuous vs. cyclical TIP treatment). Differences between groups were evaluated by multivariate-permutational analysis (permutational multivariate ANOVA as implemented within the ADONIS function of the vegan-package in R). Correlation between variables was made by calculating the Spearman's correlation coefficient, both before and after adjusting for repeated measures. A co-occurrence network construction and analysis were performed using a simplified OTU-count table (top 30% of OTUs were retained for network analysis) using the NetCoMi (ver. 1.1.0) [25] and SpiecEasi estimation (Sparse InversE Covariance estimation for Ecological Association and Statistical Inference). Where appropriate, p-values were adjusted for multiple testing using the Benjamini-Hochberg (BH) method for false-discovery rate.

A p-value <0.05 was considered statistically significant.

Metadata

The metadata can be found in Supplementary file 1.

Additional Results

Sensitivity analysis

A sensitivity analysis was performed using alternative reductions in *P. aeruginosa* density (1Log_{10} and 3Log_{10} *oprL* copies/mL) for stratifying participants as responders and non-responders. A small number of study participants changed cluster (responder vs. non-responder) with the alternative cut-offs (34 ≥1Log_{10} vs. 29 <1Log_{10}; 26 ≥2Log_{10} vs. 37 <2Log_{10}; 18 ≥3Log10 vs. 45 <3Log_{10}).

Various microbiota metrics [total bacterial density, *P. aeruginosa* density (oprL copies/mL), Pseudomonas RA (%) and *P. aeruginosa* total viable count (CFU/g), alpha-diversity] were then compared between time-points for both groups. The same trends were observed as per the 2Log_{10} cut-off used in the study.

Furthermore, we compared FEV_1 %Predicted at Day 1 (co-variate which predicted response to treatment in study) between those that responded to treatment and those that didn't based on the alternative cut-offs. Lung function was considerably higher in the responder group at Day 1 regardless of the cut-off value used: responders vs. non-responders, 1Log_{10}: 59.70 %Predicted vs. 51.31 %Predicted (p=0.140); 2Log_{10}: 64.6 %Predicted vs. 50.3 %Predicted (p=0.005); 3Log_{10}: 65.44 %Predicted vs. 52.64 %Predicted (p=0.009).

ANCOMBC (Analysis of Composition of Microbiomes with Bias Correction)

To complement our observations from the co-occurrence network analysis, we performed ANCOMBC (Analysis of Composition of Microbiomes with Bias Correction) with zero detection enabled (strc_zero=TRUE, neg_lb=FALSE) to identify taxa showing significant differences, or log-fold change (LFC, with p-values corrected for multi-test adjustment), in abundance between time points (Day 1 vs. Day 29) in the TIP groups. This method addresses common microbiome data analysis challenges, such as compositionality and sequencing depth biases.

In the responder cluster, a key finding was that significant shifts in microbial composition between Day 1 and Day 29 were apparent. This included significant increases in *Bulleidia* (LFC: 3.911, padj: <0.0001), *Rothia* (LFC: 3.507, padj: <0.0001), and *Prevotella* (LFC: 3.123, padj: <0.0001) over the 28-day treatment period. Conversely, significant decreases were observed in *Pseudomonas* (LFC: -5.45, padj: <0.0001), *Neisseria* (LFC: -2.344, padj: $1.13*10^{-4}$), and *Haemophilus* (LFC: -2.284, padj: $2.54*10^{-4}$).

For the non-responder cluster, significant increases were observed in *Bulleidia* (LFC: 1.588, padj: $4.68*10^{-4}$), *Rothia* (LFC: 1.504, padj: 0.018), and *Capnocytophaga* (LFC: 1.083, padj: 0.003). Significant decreases were observed in *Selenomonas* (LFC: -0.678, padj: 0.007), *Lautropia* (LFC: -0.801, padj: 0.013), *Campylobacter* (LFC: -0.955, padj: <0.0001), fam. Neisseriaceae Uncl. (LFC: -1.542, padj: $3.75*10^{-4}$), *Actinomyces* (LFC: -1.615, padj: 0.002), *Haemophilus* (LFC: -2.773, padj: <0.0001), and *Neisseria* (LFC: -3.044, padj: <0.0001).

In summary, the result from ANCOMBC analysis fundamentally corresponded to the observed direction of change from the co-occurrence analysis networks for responders vs. non-responders during the first 28-days of TIP treatment. The full list of differences in taxa between time points (Day 1 vs. Day 29) in the two TIP response clusters is shown in Supplementary table 2.

Online Supplement references

1. Loebinger MR, Polverino E, Chalmers JD, Tiddens HA, Goossens H, Tunney M, Ringshausen FC, Hill AT, Pathan R, Angyalosi G. Efficacy and safety of TOBI Podhaler® in Pseudomonas aeruginosa-infected bronchiectasis patients: iBEST study. *European Respiratory Journal* 2020.

2. Nadkarni MA, Martin FE, Jacques NA, Hunter N. Determination of bacterial load by real-time PCR using a broad-range (universal) probe and primers set. *Microbiology* 2002: 148(1): 257-266.

3. Deschaght P, De Baere T, Van Simaey L, Van Daele S, De Baets F, De Vos D, Pirnay JP, Vaneechoutte M. Comparison of the sensitivity of culture, PCR and quantitative real-time PCR for the detection of *Pseudomonas aeruginosa* in sputum of cystic fibrosis patients. *BMC Microbiol* 2009: 9: 244.

4. Mangiaterra G, Amiri M, Di Cesare A, Pasquaroli S, Manso E, Cirilli N, Citterio B, Vignaroli C, Biavasco F. Detection of viable but non-culturable *Pseudomonas aeruginosa* in cystic fibrosis by qPCR: a validation study. *BMC Infect Dis* 2018: 18(1): 701.

5. Lundberg DS, Yourstone S, Mieczkowski P, Jones CD, Dangl JL. Practical innovations for high-throughput amplicon sequencing. *Nat Methods* 2013: 10(10): 999-1002.

6. Caporaso JG, Kuczynski J, Stombaugh J, Bittinger K, Bushman FD, Costello EK, Fierer N, Peña AG, Goodrich JK, Gordon JI. QIIME allows analysis of high-throughput community sequencing data. *Nature methods* 2010: 7(5): 335-336.

7. Air GM, Els MC, Brown LE, Laver WG, Webster RG. Location of antigenic sites on the three-dimensional structure of the influenza N2 virus neuraminidase. *Virology* 1985: 145(2): 237-248.

8. Edgar RC. Search and clustering orders of magnitude faster than BLAST. *Bioinformatics* 2010: 26(19): 2460-2461.

9. Wang Q, Garrity GM, Tiedje JM, Cole JR. Naive Bayesian classifier for rapid assignment of rRNA sequences into the new bacterial taxonomy. *Appl Environ Microbiol* 2007: 73(16): 5261-5267.

10. DeSantis TZ, Hugenholtz P, Larsen N, Rojas M, Brodie EL, Keller K, Huber T, Dalevi D, Hu P, Andersen GL. Greengenes, a chimera-checked 16S rRNA gene database and workbench compatible with ARB. *Appl Environ Microbiol* 2006: 72(7): 5069-5072.

11. McDonald D, Price MN, Goodrich J, Nawrocki EP, DeSantis TZ, Probst A, Andersen GL, Knight R, Hugenholtz P. An improved Greengenes taxonomy with explicit ranks for ecological and evolutionary analyses of bacteria and archaea. *The ISME journal* 2012: 6(3): 610.

12. Watt A, Brown V, Courtney J, Kelly M, Garske L, Elborn J, Ennis M. Neutrophil apoptosis, proinflammatory mediators and cell counts in bronchiectasis. *Thorax* 2004: 59(3): 231-236.

13. McMurdie PJ, Holmes S. phyloseq: an R package for reproducible interactive analysis and graphics of microbiome census data. *Plos One* 2013: 8(4): e61217.

14. Lahti L, Shetty S. Introduction to the microbiome R package. *Preprint at https://microbiome github io/tutorials* 2018.

15. Xu S, Yu GM. an R package for analysis, visualization and biomarker discovery of microbiome. *R package version* 2021: 1(1).

16. Jari Oksanen FGB, Michael Friendly, Roeland Kindt, Pierre Legendre, Dan McGlinn, Peter R. Minchin, R. B. O'Hara, Gavin L. Simpson, Peter Solymos, M. Henry H. Stevens, Eduard Szoecs, Helene Wagner. vegan: Community Ecology Package. R package version 2.5-4. *https://CRANR-projectorg/package=vegan* 2019.

17. Hadley Wickham RF, Lionel Henry, Kirill Müller dplyr: A Grammar of Data Manipulation. *R package version 0801* 2019: https://CRAN.R-project.org/package=dplyr.

18. Wickham H. ggplot2: Elegant Graphics for Data Analysis. *Springer-Verlag New York* 2016.

19. Kassambara A. ggpubr:'ggplot2'based publication ready plots. *R package version* 2018: 2.

20. Patil I. Visualizations with statistical details: The'ggstatsplot'approach. *Journal of Open Source Software* 2021: 6(61): 3167.

21. Wilke CO, Wiernik BM. ggtext: Improved text rendering support for ‘ggplot2’. *R package version 01* 2020: 1.

22. Harrell Jr FE, Harrell Jr MFE. Package ‘Hmisc’. *CRAN2018* 2015: 235-236.

23. Wickham H. Reshaping data with the reshape package. *Journal of Statistical Software* 2007: 21(12).

24. Bakdash JZ, Marusich LR. Repeated measures correlation. *Frontiers in psychology* 2017: 8: 456.

25. Peschel S, Müller CL, Von Mutius E, Boulesteix A-L, Depner M. NetCoMi: network construction and comparison for microbiome data in R. *Briefings in bioinformatics* 2021: 22(4): bbaa290.

Supplementary table 1 Day 1 demographic characteristics, microbiota metrics and inflammatory biomarker profile of Responders (n=26) and non-Responders (n=37).

		Total (n=63)	Resonders: ≥2Log10 reduction (n=26)	Non-responders: <2Log10 reduction (n=37)	p-value*
**Age (median; [IQR])**		67 [58-74]	67.5 [60.0-73.8]	65 [54-74]	0.480
**Age Group (n; [%])**	**>=65**	36 [57.1]	17 [65.4]	19 [51.4]	
	**<65**	27 [42.9]	9 [34.6]	18 [48.6]	0.396
**Gender (n; [%])**	**F**	39 [61.9]	16 [61.5]	23 [62.2]	
	**M**	24 [38.1]	10 [38.5]	14 [37.8]	1.000
**FEV_1 %Predicted (median; [IQR])**		55.1 [44.3-72.4]	64.6 [52.6-84.3]	50.3 [36.5-62.6]	0.005
**Min-Max**		24-135.1	32.4-111.6	24-135.1	
**FVC %Predicted (median; [IQR])**		70.7 [57.7-90.9]	75.8 [64.7-98.4]	65.7 [56.5-88.1]	0.072
**Min-Max**		33-135.1	48.3-124.8	33-135.1	
**Microbiota metrics**					
**16S rRNA (Log10; copies/mL) (median; [IQR])**		9.3 [8.7, 9.6]	9.2 [8.8, 9.6]	9.4 [8.7, 9.7]	0.573
**Min-Max**		8-10.3	8.2-9.9	8-10.3	
***oprL* (Log10; copies/mL) (median; [IQR])**		7.9 [7.5, 8.4]	7.9 [7.7, 8.2]	7.9 [7.5, 8.4]	0.272
**Min-Max**		4-8.8	6.6-9	4-8.8	
***ecfX* (Log10; copies/mL) (median; [IQR])**		7.9 [7.3, 8.3]	7.9 [7.7, 8.3]	7.9 [6.9, 8.3]	0.097
**Min-Max**		4-9.1	6.6-9.1	4-9.1	
***P. aeruginosa* (Log10; CFU/g sputum) (median; [IQR])**		6.8 [5.6, 7.8]	7 [6.4, 7.6]	7 [5.9, 7.7]	0.198
**Min-Max**		2-10.2	5.6-9	2-10.2	
***Pseudomonas* (%RA) (median; [IQR])**		56.6 [22.6, 88.5]	64.3 [24.0, 88.4]	61 [22.5, 88.5]	0.397
**Min-Max**		0-98.8	11.1-97.1	0-98.8	
**Shannon-Wiener Index (H) (median; [IQR])**		1 [0.5, 1.6]	0.9 [0.6, 1.5]	1 [0.5, 1.6]	0.811
**Min-Max**		0.1-2.5	0.2-2.7	0.1-2.7	
**Dominance (D) (median; [IQR])**		0.5 [0.3, 0.8]	0.6 [0.4, 0.8]	0.5 [0.4, 0.8]	0.968
**Min-Max**		0.1-1	0.1-0.9	0.1-1	
**Inflammatory biomarkers: blood**					
**CRP (mg/l) (median; [IQR])**		8.8 [2.8, 27.3]	10.9 [4.7, 27.3]	5.5 [2.4, 23.9]	0.247
**Min-Max**		0.4-81.3	0.8-81.3	0.4-55	
**Eosinophils ($x10^9$/L) (median; [IQR])**		0.1 [0.01, 0.2]	0.1 [0.1, 0.2]	0.2 [0.01, 0.2]	0.243
**Min-Max**		0-1.7	0-0.6	0-1.7	
**Inflammatory biomarkers: sputum**					
**IL8 (pg/mL) (median; [IQR])**		9,715.9 [5,254.6, 17,391.6]	12,389.9 [8,285.5, 17,439.7]	11,336.8 [6,223.5, 17,942.0]	0.409
**Min-Max**		859.8-37,863.3	1,509.3-44,656.8	859.8-44,656.8	
**Neutrophil elastase (ng/mL) (median; [IQR])**		3,741.4 [2,615.8, 5,480.0]	3,546.1 [2,589.8, 6,578.3]	3,670 [2,581.8, 6,073.2]	0.826
**Min-Max**		2,274.8-159,661.8	1,289.3-97,872.6	1,289.3-159,661.8	
**IL1b (pg/mL) (median; [IQR])**		1,124.1 [709.3, 2,227.8]	747.5 [341.9, 2,466.3]	910.1 [362.0, 2,454.8]	0.350
**Min-Max**		35.6-5,234.9	82.9-10,345	35.6-10,345	
**HMGB1 (ng/mL) (median; [IQR])**		0.4 [0.4, 1.6]	0.4 [0.4, 8.5]	0.4 [0.4, 2.8]	0.466
**Min-Max**		0.4-93	0.4-88.5	0.4-93	
**Calprotectin (ng/mL) (median; [IQR])**		28,187.8 [11,561.5, 44,837.3]	27,222.4 [9,649.3, 43,147.6]	28,187.8 [9,816.3, 43,674.5]	0.973
**Min-Max**		81.8-101,801.7	540.7-90,927.3	81.8-101,801.7	

Definitions: IQR, interquartile range; FEV_1 %Predicted, forced expiratory volume in one-second %Predicted; IL, interleukin; HMGB1, high mobility group box 1 protein; CRP, C-reactive protein; CI, confidence interval.

*Statistical comparisons made between responders and non-responders; Wilcoxon test; $p<0.05$ denotes statistical significance.

Supplementary table 2 ANCOMBC analysis of differences in taxa between time-points (Day 1 vs. Day 29) in responder and non-responder clusters.

	Taxa	LFC	se	W	p-value	p-value adjusted
**Responders**	**Bulleidia**	3.911	0.419	9.339	<0.0001	<0.0001
	**Rothia**	3.507	0.406	8.635	<0.0001	<0.0001
	**Prevotella**	3.123	0.453	6.897	<0.0001	<0.0001
	**Porphyromonas**	2.388	0.633	3.774	0.0002	0.0074
	**Capnocytophaga**	2.325	0.394	5.9	<0.0001	<0.0001
	**Paraprevotella**	2.123	0.531	4.001	<0.0001	0.0031
	**fam. Lachnospiraceae Uncl.**	1.98	0.541	3.664	0.0002	0.0107
	**Bifidobacterium**	1.854	0.43	4.31	<0.0001	0.0009
	**Scardovia**	1.821	0.377	4.827	<0.0001	<0.0001
	**fam. Bifidobacteriaceae Uncl.**	1.694	0.422	4.012	<0.0001	0.0030
	**fam. Weeksellaceae Uncl.**	1.694	0.346	4.89	<0.0001	<0.0001
	**Streptococcus**	1.479	0.364	4.06	<0.0001	0.0026
	**Tannerella**	1.435	0.295	4.867	<0.0001	<0.0001
	**cla. Clostridiales Uncl.**	0.906	0.202	4.492	<0.0001	0.0004
	**Treponema**	0.866	0.246	3.515	0.0004	0.0184
	**TG5**	0.814	0.202	4.018	<0.0001	0.0030
	**Dialister**	0.776	0.201	3.865	0.0001	0.0053
	**Shuttleworthia**	0.754	0.23	3.284	<0.0001	<0.0001
	**Pseudoramibacter_Eubacterium**	0.748	0.228	3.276	0.0011	0.0419
	**fam. Ruminococcaceae Uncl.**	0.744	0.226	3.284	0.0010	0.0419
	**fam. Clostridiaceae Uncl.**	0.69	0.185	3.728	0.0002	0.0085
	**Kingella**	0.165	0.191	0.865	<0.0001	<0.0001
	**Lautropia**	-0.042	0.249	-0.167	<0.0001	<0.0001
	**Aggregatibacter**	-0.167	0.257	-0.65	<0.0001	<0.0001
	**fam. Neisseriaceae Uncl.**	-0.982	0.262	-3.742	0.0002	0.0082
	**Granulicatella**	-1.308	0.345	-3.789	0.0002	0.0071
	**Haemophilus**	-2.284	0.498	-4.582	<0.0001	0.0003
	**Neisseria**	-2.344	0.493	-4.751	<0.0001	0.0001
	**fam. Pseudomonadaceae Uncl.**	-2.926	0.268	-10.91	<0.0001	<0.0001
	**Pseudomonas**	-5.45	0.446	-12.213	<0.0001	<0.0001
**non-Responders**	**Bulleidia**	1.588	0.356	4.463	<0.0001	<0.0001
	**Rothia**	1.504	0.420	3.577	0.0018	0.0035
	**Capnocytophaga**	1.083	0.267	4.064	<0.0001	<0.0001
	**fam. Pasteurellaceae Uncl.**	-0.052	0.148	-0.356	<0.0001	<0.0001
	**Cardiobacterium**	-0.218	0.170	-1.284	<0.0001	<0.0001
	**Kingella**	-0.249	0.158	-1.583	<0.0001	<0.0001
	**Selenomonas**	-0.678	0.177	-3.842	0.0067	0.0100
	**Lautropia**	-0.801	0.218	-3.672	0.0001	0.0002
	**Campylobacter**	-0.955	0.192	-4.974	<0.0001	<0.0001
	**fam. Neisseriaceae Uncl.**	-1.542	0.342	-4.514	<0.0001	<0.0001
	**Actinomyces**	-1.615	0.384	-4.208	<0.0001	<0.0001
	**Haemophilus**	-2.773	0.535	-5.188	<0.0001	<0.0001
	**Neisseria**	-3.044	0.465	-6.553	<0.0001	<0.0001

Definitions: LFC, log-fold change; se, standard error; W, test statistic.

Supplementary table 3 Day 1 variables and their association with being a responder.

	Responders (n=26)	Non-responders (n=37)	Odds ratio (95% CI)†	p-value
**FEV_1 %Predicted (median; [IQR])**	64.6 (52.6-84.3)	50.3 (36.5-62.6)	1.035 (1.007-1.064)	0.013*
**Number of pulmonary exacerbations in the previous 12-months (median; [IQR])**	2.5 (2-3)	2 (2-3)	1.578 (0.987-2.524)	0.057*
**Microbiota metrics**				
**Shannon Wiener diversity [H] (median; [IQR])**	0.88 (0.56-1.50)	0.99 (0.54-1.57)	1.138 (0.531-2.438)	0.739
**Log_{10} total 16S rRNA (copies/mL) (median; [IQR])**	9.2 (8.8-9.6)	9.4 (6.7-9.7)	0.800 (0.326-1.962)	0.626
**Log_{10} *Pseudomonas aeruginosa* (*oprL* copies/mL) (median; [IQR])**	7.9 (7.7-8.2)	7.9 (7.5-8.4)	1.430 (0.821-2.493)	0.207
**Inflammatory Biomarkers**				
**Log_{10} elastase (ng/mL) (median; [IQR])**	3.5 (3.4-3.8)	3.6 (3.4-3.7)	0.837 (0.250-2.804)	0.773
**Log_{10} IL8 (pg/mL) (median; [IQR])**	4.1 (3.9-4.2)	4.0 (3.7-4.2)	1.604 (0.400-6.422)	0.505
**Log_{10} IL1b (pg/mL) (median; [IQR])**	2.9 (2.5-3.4)	3.1 (2.9-3.3)	0.927 (0.347-2.473)	0.880
**Log_{10} HMGB1 (ng/mL) (median; [IQR])**	0.14 (0.14-0.95)	0.14 (0.14-0.40)	1.737 (0.693-4.355)	0.239
**Log_{10} calprotectin (ng/mL) (median; [IQR])**	4.4 (4.0-4.6)	4.5 (4.1-4.7)	0.864 (0.319-2.342)	0.774
**Eosinophils ($x10^9$/L) (median; [IQR])** ‡	0.14 (0.01-0.21)	0.17 (0.11-0.24)	0.159 (0.004-6.318)	0.327
**CRP (mg/L) (median; [IQR])**	10.9 (4.7-27.3)	5.5 (2.4-23.9)	1.018 (0.987-1.050)	0.262

†Odds of being a responder compared to being a non-responder are provided (adjusted for age and gender). Variables were analysed per unit increase.

*Both FEV_1 %predicted and number of pulmonary exacerbations in the previous 12-months were included in the multivariable model but only lung function remained statistically significant. Final model fit: Hosmer and Lemeshow Test, χ^2=7.617, p=0.472; Nagelkerke R^2=0.16.

Definitions: IQR, interquartile range; FEV_1 %predicted, forced expiratory volume in one-second %predicted; IL, interleukin; HMGB1, high mobility group box 1 protein; CRP, C-reactive protein; CI, confidence interval.

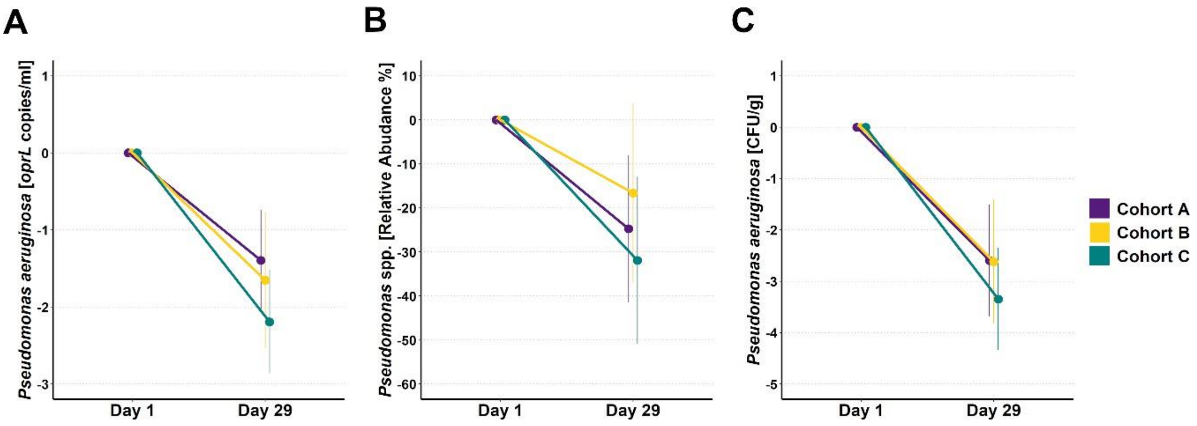

Supplementary figure 1 Change in *Pseudomonas aeruginosa* density/*Pseudomonas* relative abundance from Day 1 to Day 29 for the tobramycin inhalation powder treatment cohorts. (A) *P. aeruginosa* density (*oprL* copies/mL) (B) *Pseudomonas* spp. (% relative abundance) and (C) *P. aeruginosa* density by culture (colony forming units per gram of sputum) (n=63).

Treatment cohorts: Cohort A = 3 capsules [O.D.; 84mg]; Cohort B = 5 capsules [O.D.; 140mg]; Cohort C = 4 capsules [B.I.D.; 224mg].

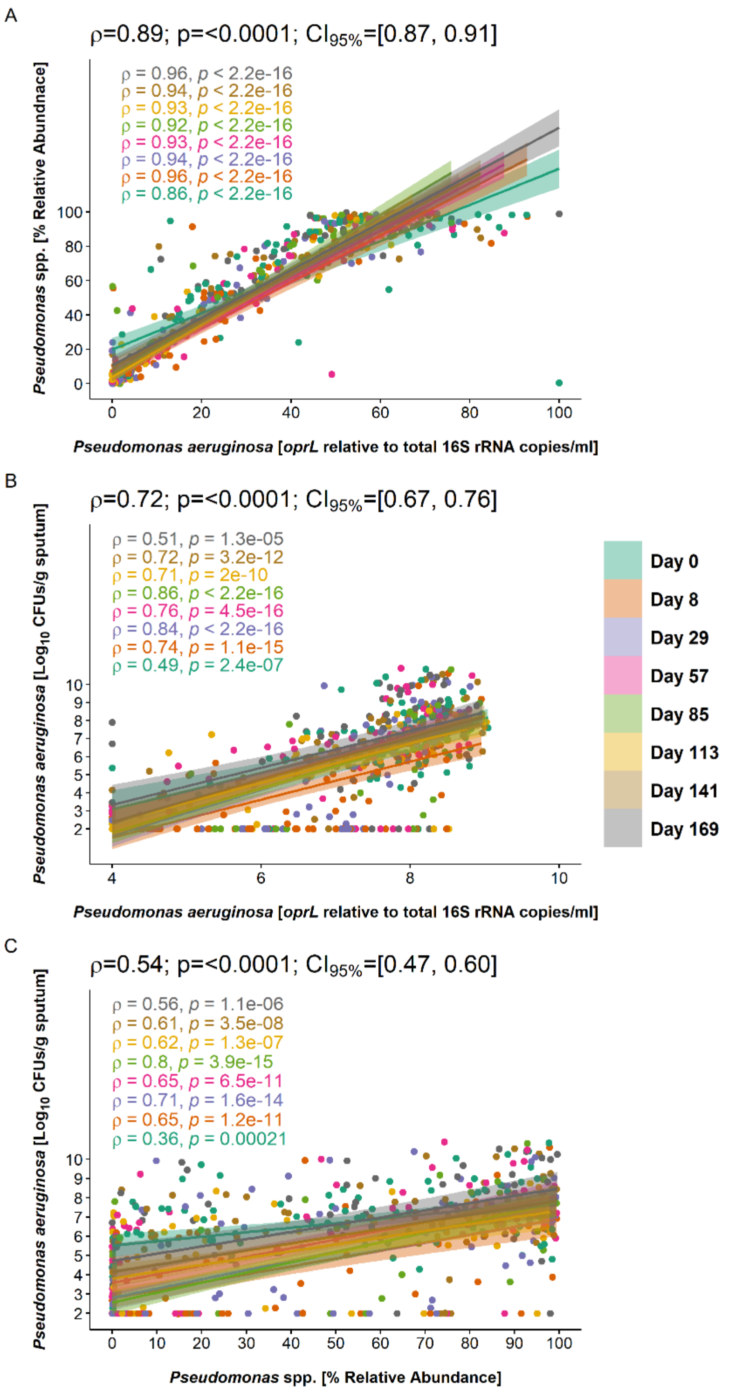

Supplementary figure 2 Correlation (ρ; Spearman's Correlation Coefficient) between culture-independent and culture-dependent methodologies in detecting *Pseudomonas* (*aeruginosa*) in clinical samples. (A) *Pseudomonas* relative abundance vs. *P. aeruginosa* (*oprL* relative adjustment by 16S rRNA density [copies/mL]). (B) Total *P. aeruginosa* density by culture (colony forming units per gram of sputum) vs. *P. aeruginosa* (*oprL* relative adjustment by 16S rRNA density [copies/mL]). (C) Total *P. aeruginosa* density by culture (colony forming units per gram of sputum) vs. *Pseudomonas* relative abundance. $p<0.05$ denotes statistically significant difference.

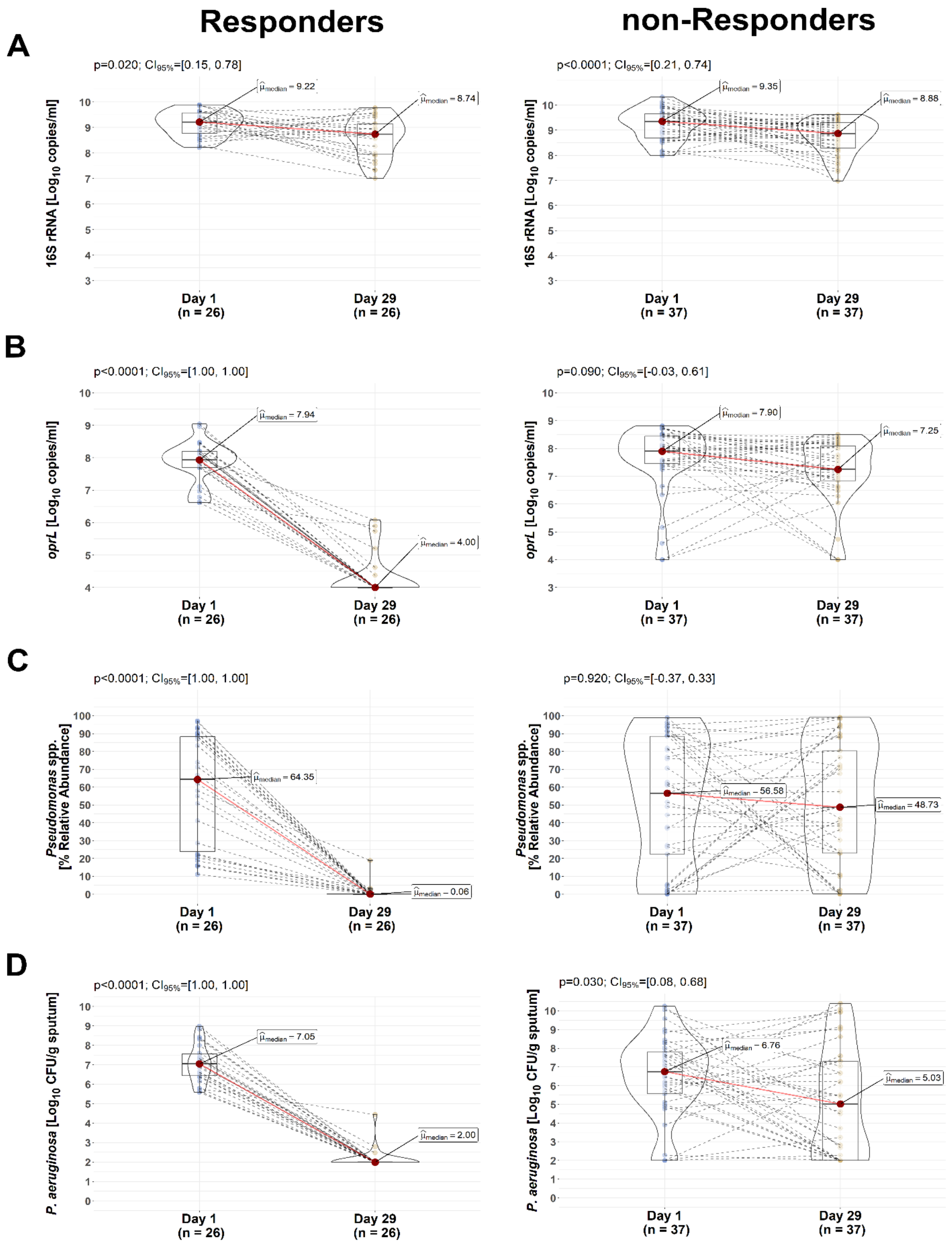

Supplementary figure 3 Comparison between Day 1 and Day 29 for participants in responder versus non- responder clusters. (A) Total bacterial density (16S rRNA copies/mL), (B) *P. aeruginosa* density (*oprL* copies/mL), (C) *Pseudomonas* spp. (% relative abundance) and (D) *P. aeruginosa* density by culture (colony forming units per gram of sputum). $p<0.05$ denotes statistically significant difference.

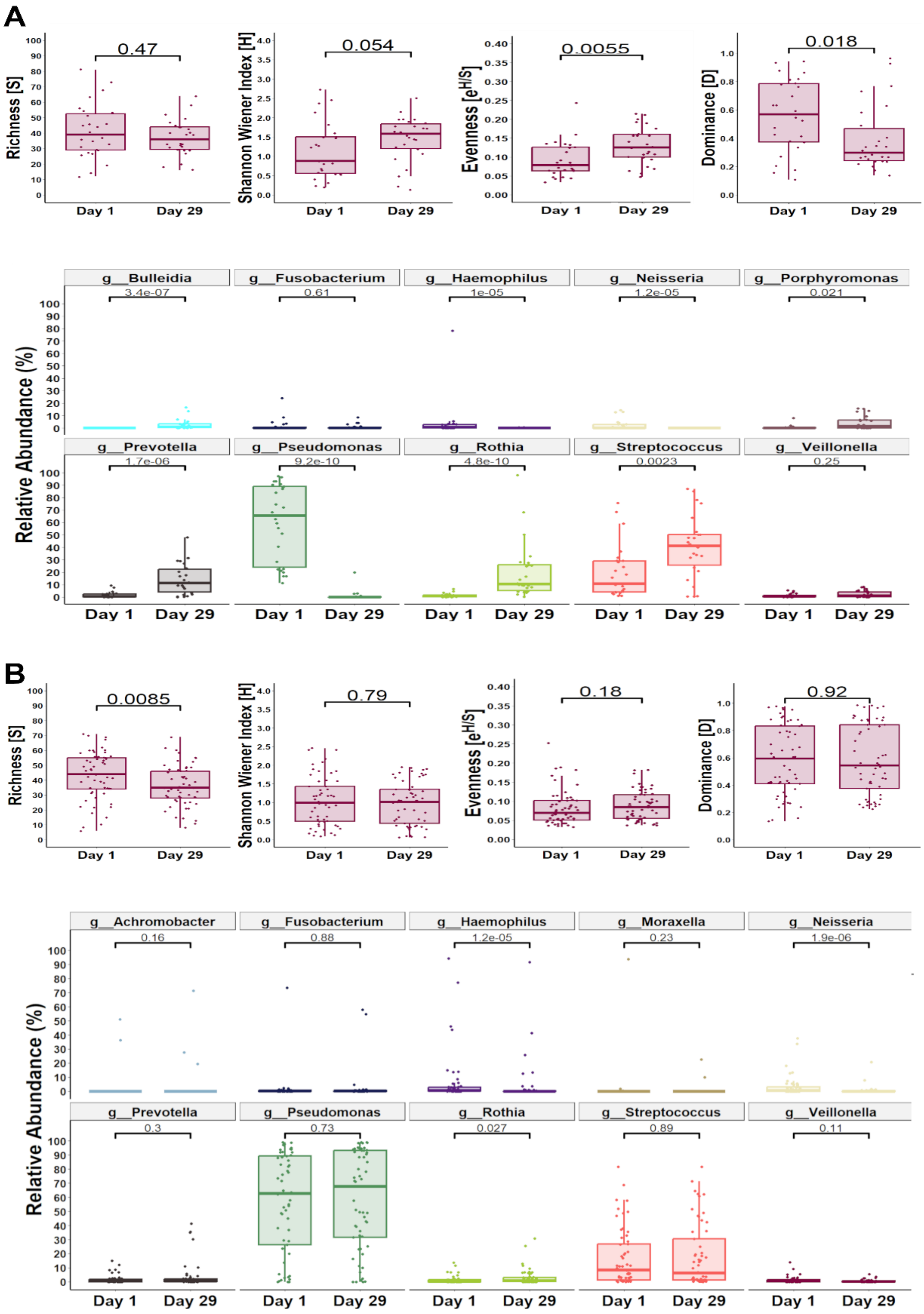

Supplementary figure 4 α-diversity metrics and relative abundance of the top 10 taxa at Day 1 and Day 29 in (A) responders and (B) non-responders to tobramycin inhalation powder treatment. $p<0.05$ denotes statistically significant difference.

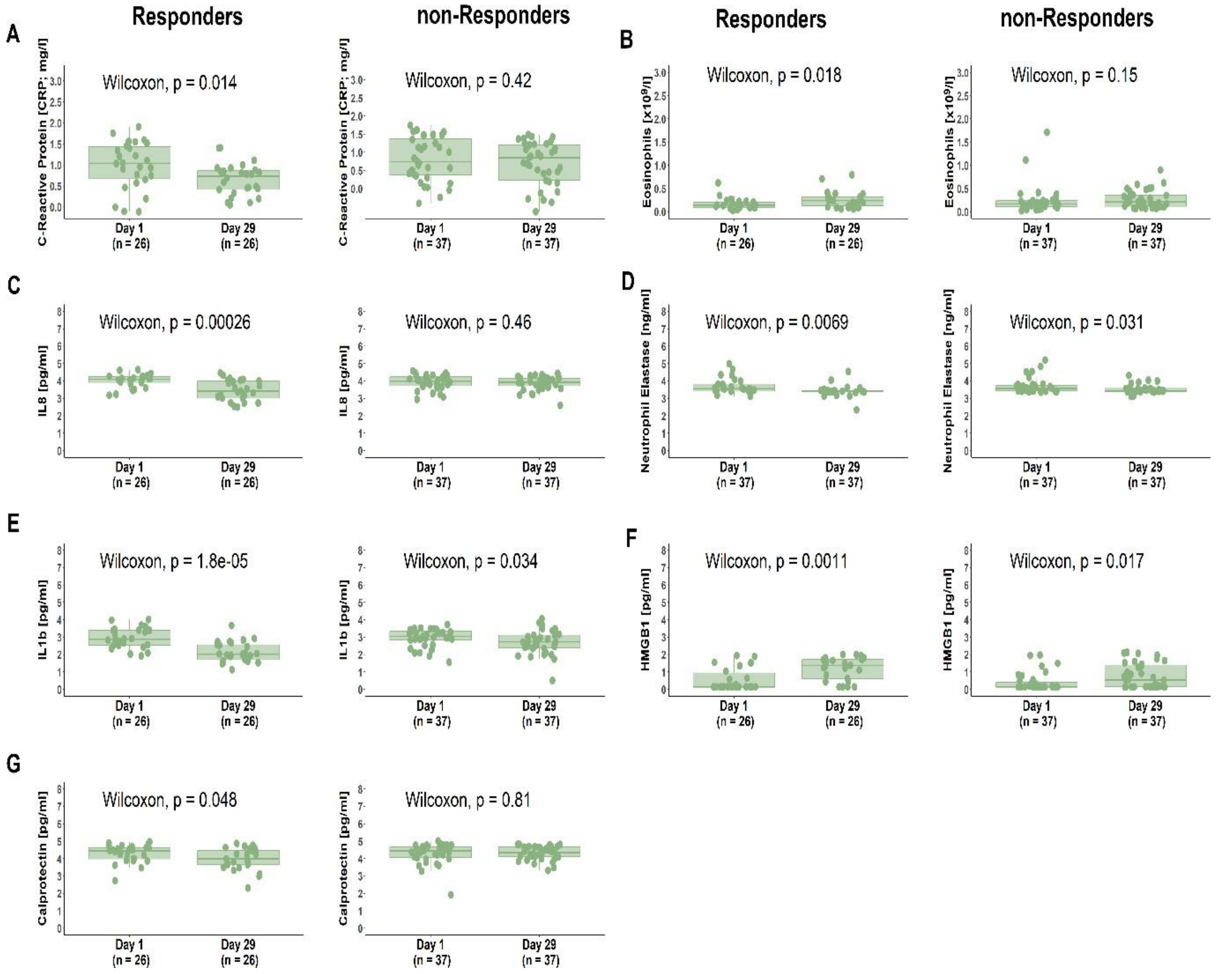

Supplementary figure 5 Inflammatory biomarker levels in responders and non-responders at Day 1 and Day 29. (A) C-Reactive Protein (CRP), (B) Eosinophil count, (C) Interleukin-8 (IL8), (D) Neutrophil Elastase, (E) Interleukin-6 (IL6), (F) High mobility group box 1 (HMGB1) and (G) Calprotectin. $p<0.05$ denotes statistical significance.

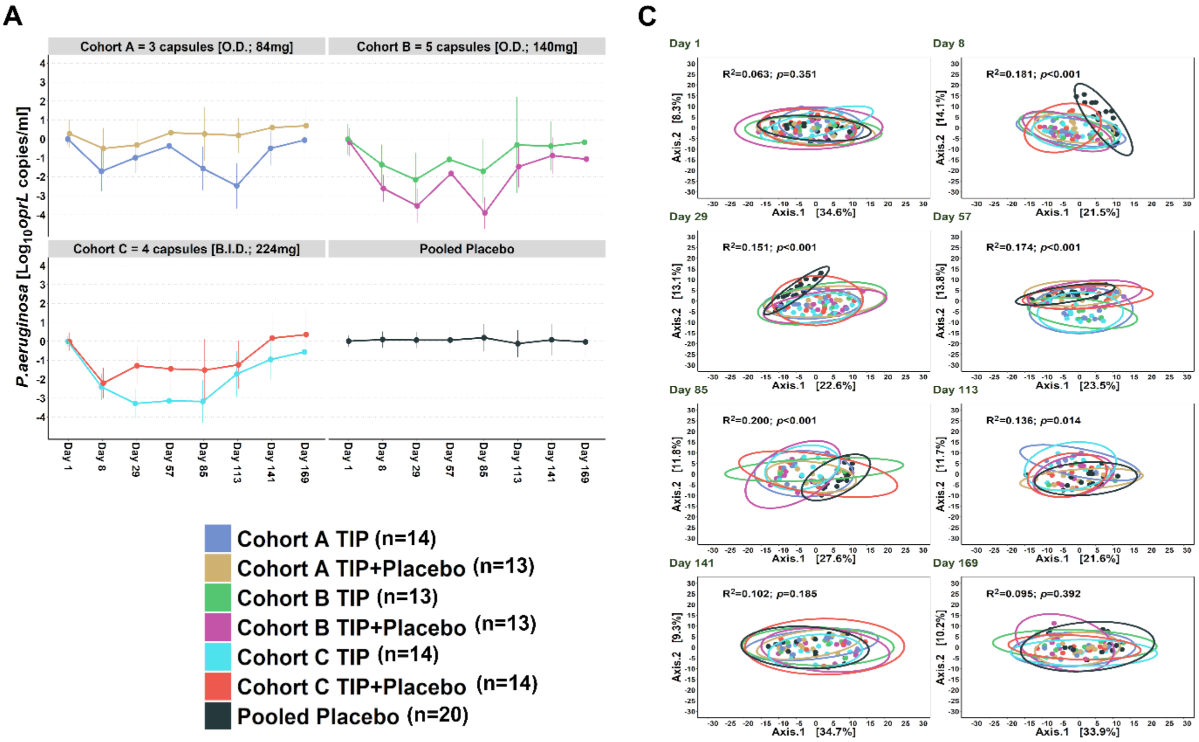

B

Cohort	Day 1	Day 8	Day 29	Day 57	Day 85	Day 113	Day 141	Day 169
Cohort A	27	21	23	22	19	18	22	20
Cohort B	26	21	19	20	13	13	17	15
Cohort C	28	23	23	20	14	15	15	13
Pooled Placebo	20	20	20	17	17	15	15	17

Supplementary figure 6 (A) Longitudinal trajectory in *Pseudomonas aeruginosa* density (*oprL* copies/mL) by visit time-point and treatment cohort (dosing: continuous vs. cyclic tobramycin inhalation powder treatment; n=101 participants with a valid Day 1 sample). (B) Number of samples available per treatment cohort and visit. Not all participants had samples available at all time-points. (C) Visualisation of canonical ordination plots (CAP), for Hellinger transformed OTU count data, based on visit time-point and treatment cohort (dosing: continuous vs. cyclic tobramycin inhalation powder treatment or pooled placebo). The different colors denote the various cohorts with comparisons based on PERMANOVA analysis (permutational multivariate ANOVA) as implemented within the ADONIS function of the vegan-package in R. $p<0.05$ denotes statistical significance and confidence (ellipses) are based on 90%CI.
