# Supplementary figures and images for: Molecular endotyping in people with bronchiectasis based on response to antibiotic treatment: iBEST study
Source: ERJ Open Res. 2025 Dec 29;11(6):00389-2025. doi: 10.1183/23120541.00389-2025 (PMC12746121; doi:10.1183/23120541.00389-2025)

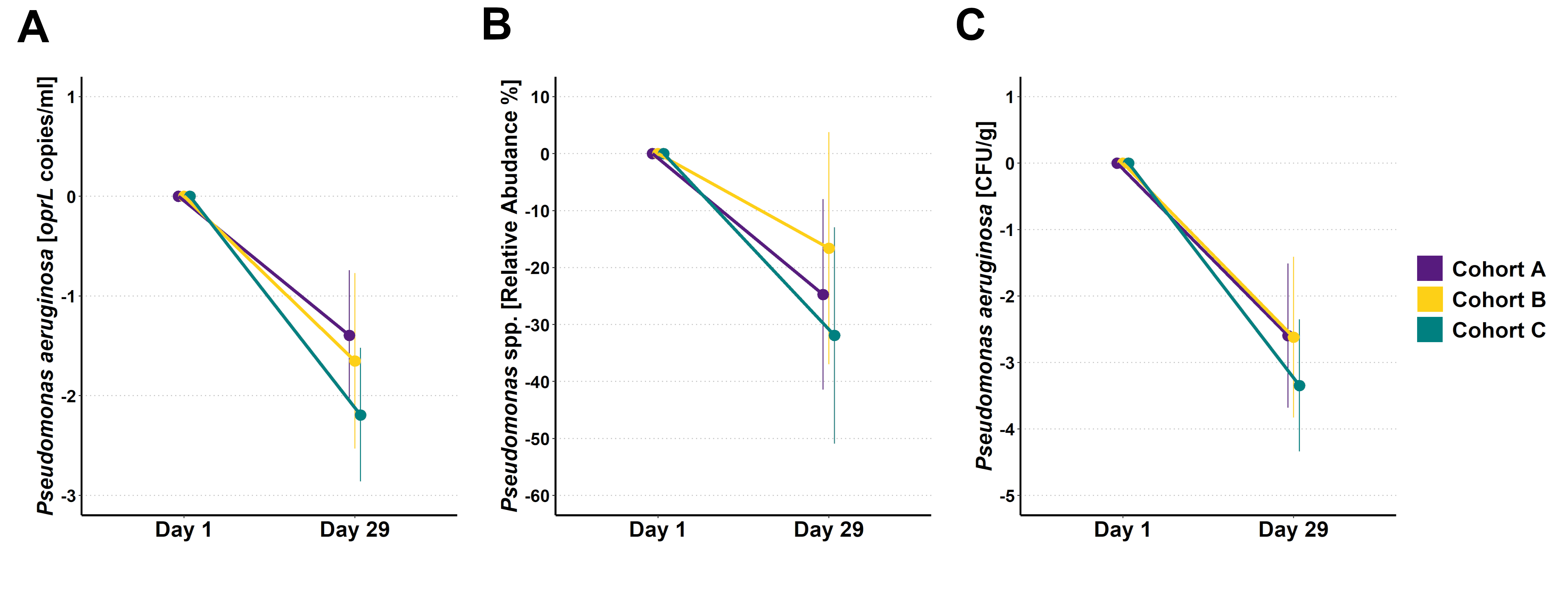

Supplement: Supplementary file 3 [file 00389-2025.SUPPLEMENT.tif]

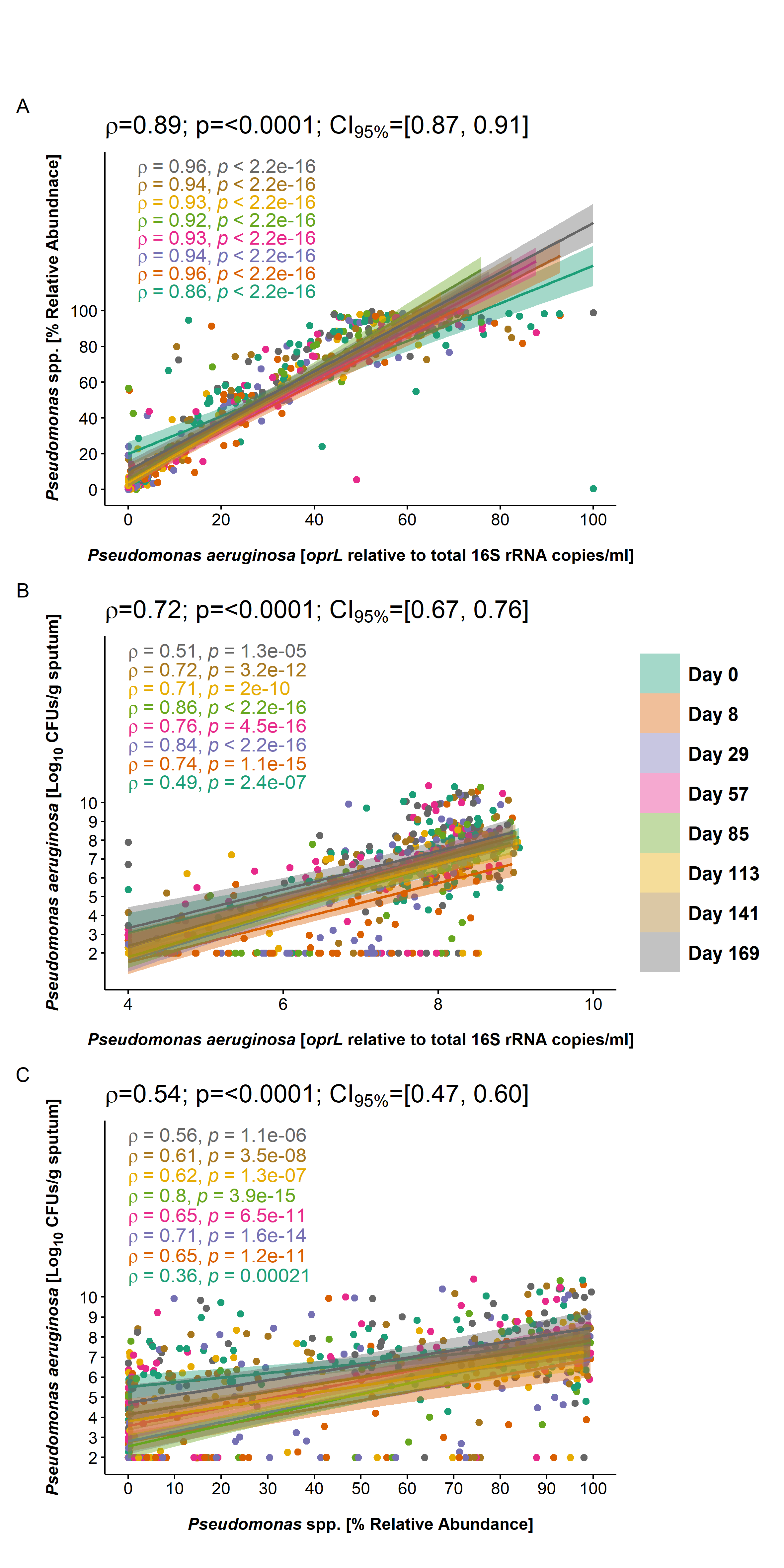

Supplement: Supplementary file 4 [file 00389-2025.SUPPLEMENT2.tif]

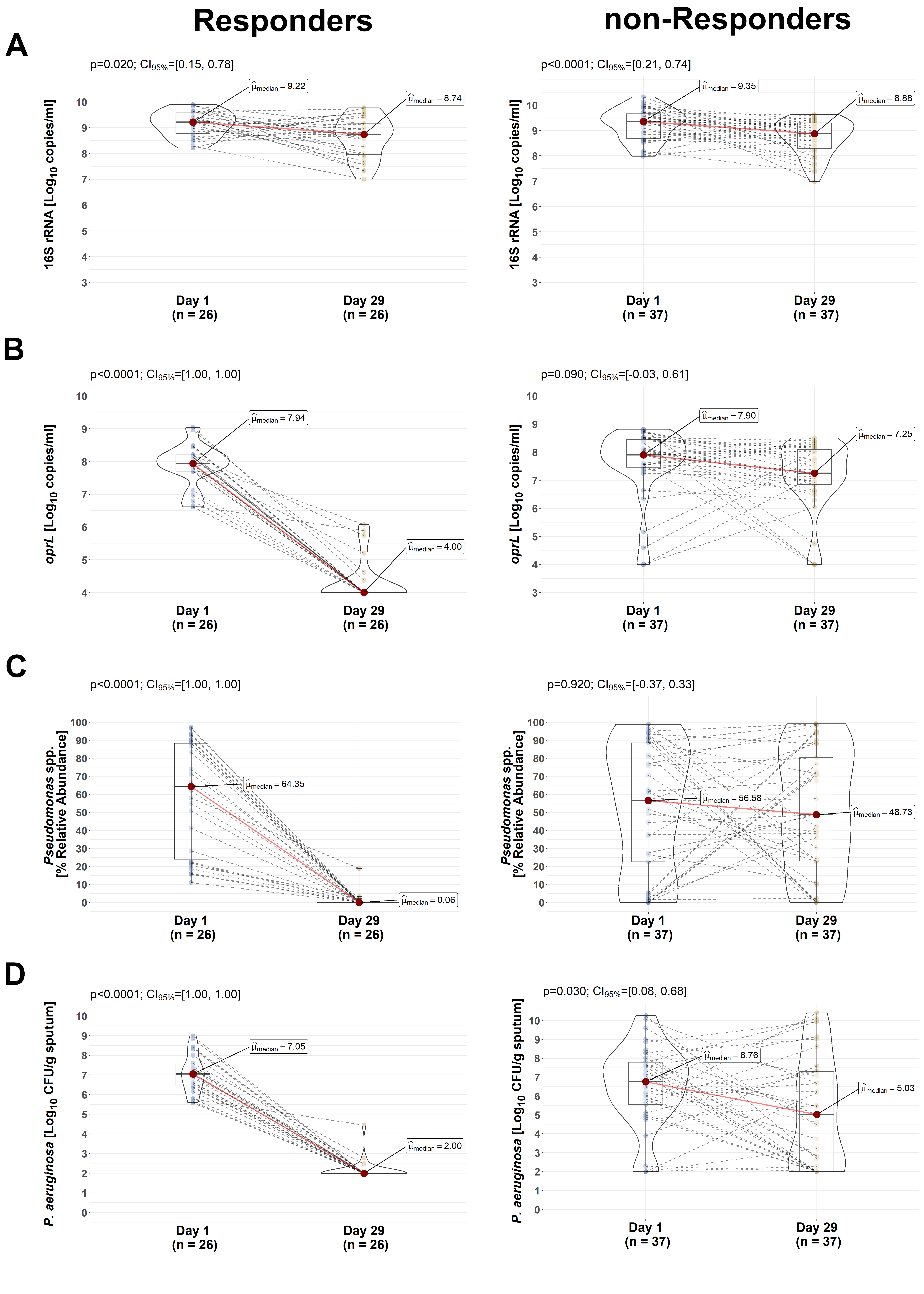

Supplement: Supplementary file 5 [file 00389-2025.SUPPLEMENT3.tif]

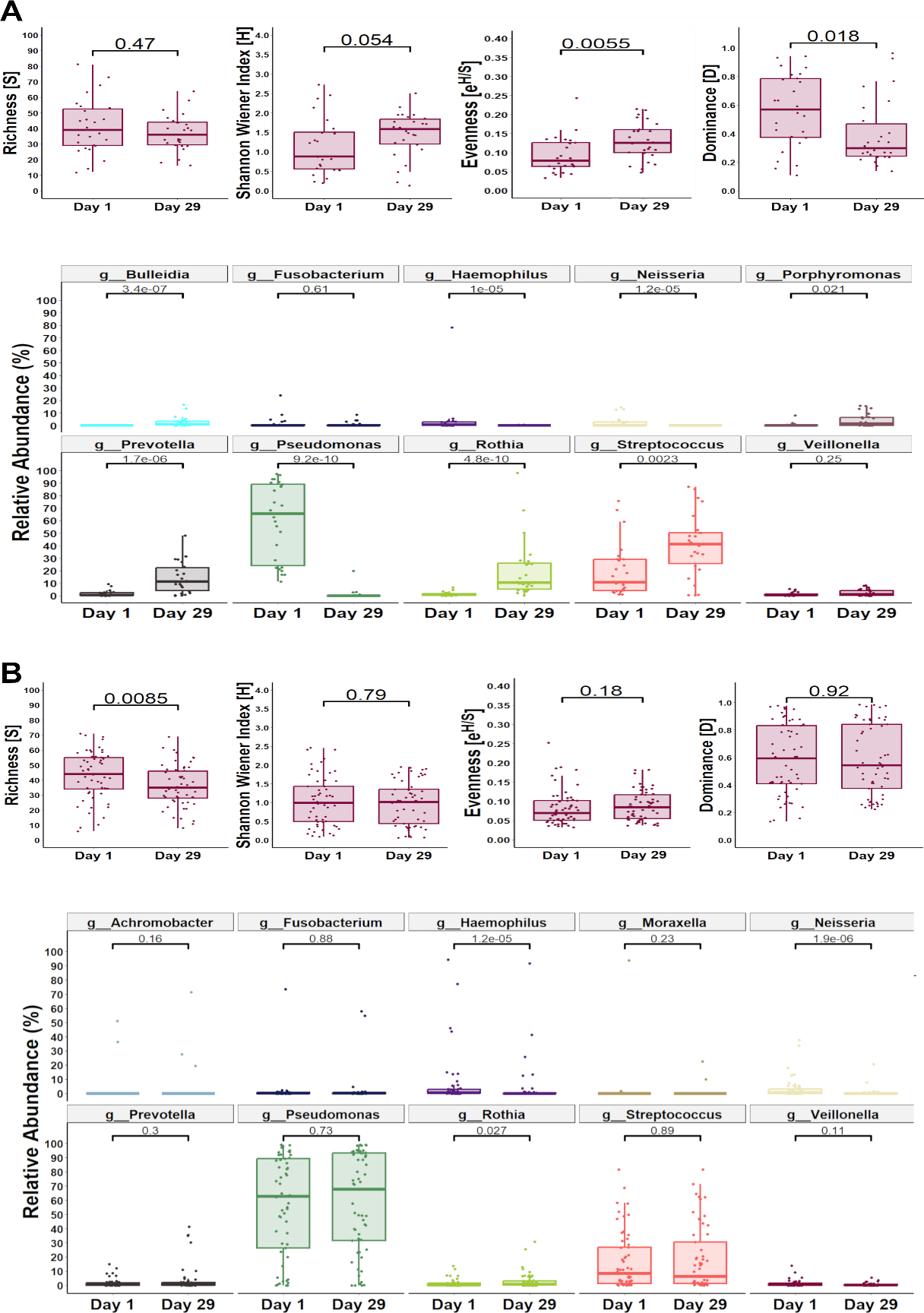

Supplement: Supplementary file 6 [file 00389-2025.SUPPLEMENT4.tif]

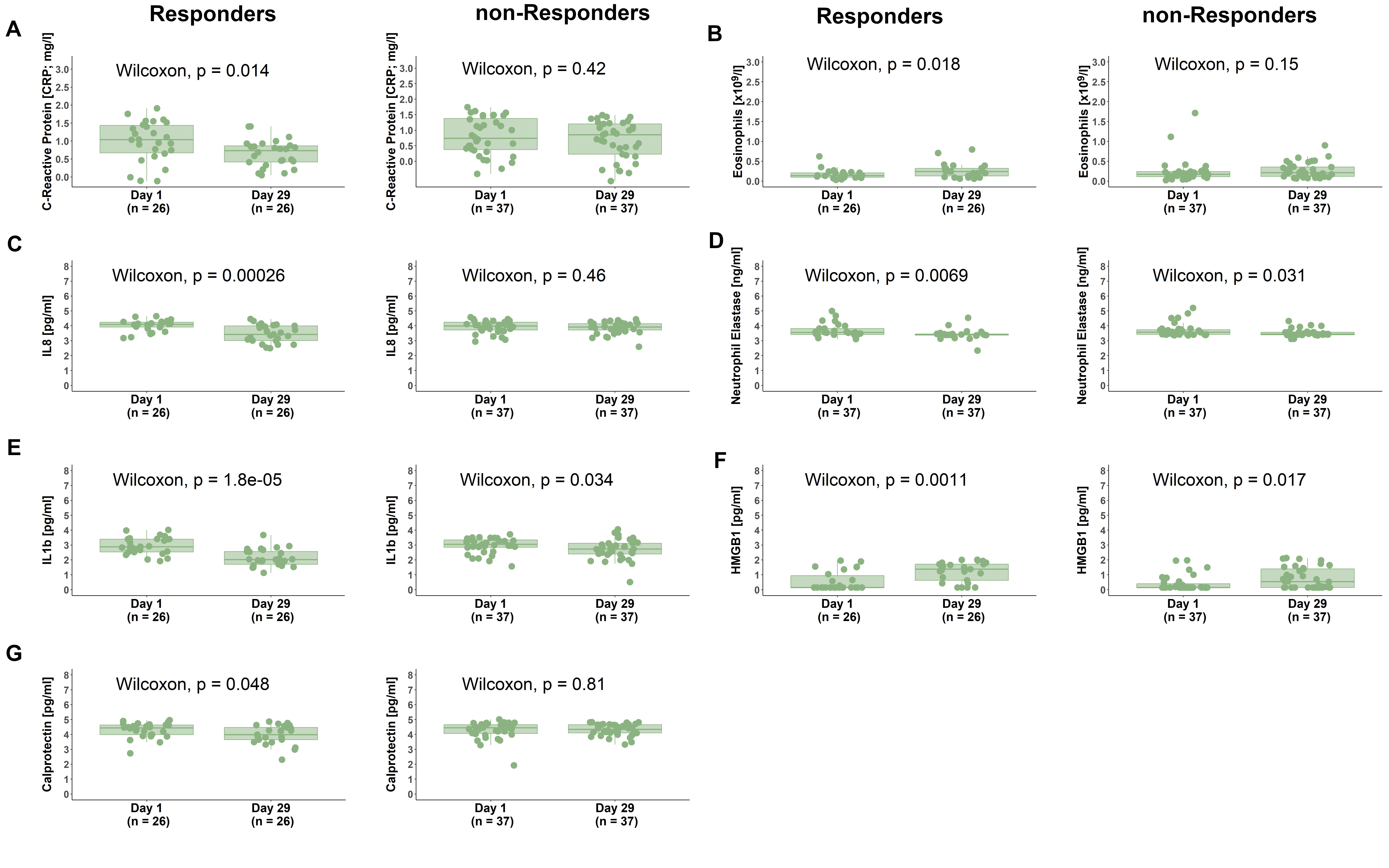

Supplement: Supplementary file 7 [file 00389-2025.SUPPLEMENT5.tif]
